# The emerging role of SPHK1 at the immune-metabolic interface: a pan-cancer integrative analysis

**DOI:** 10.1038/s41598-026-35350-7

**Published:** 2026-01-17

**Authors:** Lei Wang, Guodong Zhong, Hao Luo, Qiao He, Yan Chen, Wei Li, Qiuju Wang

**Affiliations:** 1 Department of Cardiovascular Surgery, Fujian Medical University Union Hospital, Fuzhou, China; 2 Key Laboratory of Cardio-Thoracic Surgery, Fujian Medical University, Fujian Province University, Fuzhou, China; 3 Department of Pathology, The Second Affiliated Hospital of Fujian University of Traditional Chinese Medicine, Fuzhou, China; 4 Department of Clinical Laboratory, Sichuan Clinical Research Center for Cancer, Sichuan Cancer Hospital & Institute, Sichuan Cancer Center, University of Electronic Science and Technology of China, NO. 55 South Renmin Road, Wuhou District, Chengdu, 610041 Sichuan China; 5 Department of Clinical Pharmacy, Sichuan Clinical Research Center for Cancer, Sichuan Cancer Hospital & Institute, Sichuan Cancer Center, University of Electronic Science and Technology of China, Chengdu, China; 6 Department of Vascular Intervention, The People’s Hospital of Jingmen, Jingmen, Hubei China

**Keywords:** SPHK1, Metabolic immune checkpoint, Cancer, Biomarker, Prognosis, Tumor microenvironment, Immunotherapy, Cancer, Computational biology and bioinformatics, Drug discovery

## Abstract

Cancer remains a major global health challenge, with incidence and mortality rates continuing to rise. Metabolic reprogramming, a hallmark of cancer, not only sustains rapid proliferation but also shapes an immunosuppressive tumor microenvironment. Among metabolic enzymes, sphingosine kinase 1 (SPHK1) plays a key role in sphingolipid signaling by regulating the balance between sphingosine-1-phosphate and sphingosine. This regulation influences both cell fate and immune responses. However, the role of SPHK1 as a potential “metabolic immune checkpoint” across various cancers, as well as its implications for prognosis and immunotherapy, remains insufficiently explored. In this pan-cancer study, we analyzed SPHK1 expression using RNA-seq data from The Cancer Genome Atlas, which includes 33 cancer types. We also examined its clinical association. We then validated SPHK1 expression at the mRNA and protein levels in clinical samples of head and neck squamous cell carcinoma (HNSC), stomach adenocarcinoma (STAD), and liver hepatocellular carcinoma (LIHC) using RT‑qPCR and immunohistochemistry, and assessed its effect on cancer cell viability using the CCK‑8 assay. Furthermore, we conducted integrated analyses to evaluate the relationship between SPHK1 expression and key immunological features, including immune cell infiltration, tumor mutation burden (TMB), microsatellite instability (MSI), and immune checkpoint gene expression. These analyses aimed to delineate SPHK1’s role in immune modulation. We observed significant upregulation of SPHK1 in multiple cancers, especially in HNSC, STAD, and LIHC. We also confirmed its ability to enhance cancer cell viability. High SPHK1 expression is consistently associated with poor patient survival, supporting its prognostic value. Importantly, comprehensive immunological analyses revealed that SPHK1 expression is closely linked to immunosuppressive features across cancers, including altered immune cell infiltration and elevated expression of established immune checkpoint molecules, positioning SPHK1 as a key regulator linking metabolic dysregulation to immune evasion. Our findings suggest that SPHK1 acts as an oncogene and prognostic biomarker. Additionally, it functions as a novel “metabolic immune checkpoint” across multiple cancer types. SPHK1 may bridge sphingolipid metabolism with tumor immune suppression and represents a potential promising integrated target for metabolically informed immunotherapy strategies.

**Supplementary Information:**

The online version contains supplementary material available at 10.1038/s41598-026-35350-7.

## Introduction

Since 2010, cancer has been the leading cause of death in China, marked by increasing rates of incidence, mortality, and overall impact. From 2005 to 2020, the total number of cancer-related deaths in China increased by 21.6%, reaching 2,397,772 fatalities^1^. Cancer’s extensive impact surpasses individual patients, imposing considerable financial strain on healthcare systems worldwide. As precision medicine progresses, cancer treatment has become more complex, emphasizing the need for personalized approaches. Since patients with the same cancer type exhibit significant variation in treatment response, it is urgent to identify common therapeutic targets and biomarkers that apply to multiple tumors^2^.

Tumor metabolic reprogramming is one of the core characteristics of cancer. Besides shaping an immunosuppressive tumor microenvironment and driving immune evasion, it provides energy and biological macromolecules for rapidly proliferating cells^3^. Sphingolipid metabolism is an important pathway in this context. Its homeostasis closely regulates cell survival, death, and inflammatory responses^4^. Sphingosine kinase 1 (SPHK1) serves as the key rate-limiting enzyme in this pathway, while the balance between its catalytic product, sphingosine-1-phosphate (S1P), and its substrate, sphingosine, directly determines cell fate^5^. Recent studies have revealed that the SPHK1/S1P axis is abnormally activated in various types of cancer. It not only directly drives tumor progression by promoting cell proliferation, migration, and drug resistance, but also functions as an important immune regulatory hub^6,7^. As a bioactive lipid mediator, S1P can influence immune cell trafficking, differentiation, and function and contribute to shaping a suppressive tumor immune microenvironment^8^. This suggests that SPHK1 may go beyond its traditional role as an “oncogene” and act as a “metabolic immune checkpoint” linking metabolic dysregulation in tumor cells with immunosuppressive states.

Although sporadic studies have reported the cancer-promoting functions of SPHK1 in specific cancer types^9,10^, comprehensive research systematically positioning SPHK1 as a “metabolic immune checkpoint” at the pan-cancer level is still lacking. This study aims to go beyond traditional single-molecule correlation analyses by building an SPHK1-centered multidimensional atlas. This endeavor seeks to bridge the knowledge gap between “metabolic enzyme dysregulation” and “clinical immune phenotypes,” thereby providing a novel way to integrate knowledge about how tumor metabolic reprogramming drives immune evasion.

This study systematically analyzed SPHK1 expression across multiple cancers and examined its associations with patient prognosis and clinical features, including treatment response. To elucidate the role of SPHK1 in cancer biology, especially in the immune microenvironment, we employed a combination of bioinformatics techniques, including RNA sequencing data analysis, immune infiltration assessment, co-expressed gene enrichment analysis, and tumor microenvironment dissection. Additionally, we conducted experimental validation on three distinct tumor types: HNSC, STAD, and LIHC. This confirmed the expression of SPHK1 and its impact on cell proliferation, thereby enhancing the reliability of our bioinformatics findings. This study aims to provide detailed insights into SPHK1 as a potential biomarker, a novel “metabolic immune checkpoint,” and a promising therapeutic target.

## Methods

### Expression of SPHK1 in different cancers

Gene expression data were acquired from The Cancer Genome Atlas (TCGA) via the TCGAbiolinks R package (v2.34.1), utilizing the GDCquery, GDCdownload, and GDCprepare functions^11^. Patient survival information and clinical data for the analyzed TCGA cohorts were obtained from the UCSC Xena platform (https://xenabrowser.net/datapages/). Then, the data were quantified as Transcripts Per Million (TPM). All gene expression matrices were log2-transformed (log2(TPM + 1)) and corrected for batch effects using the ComBat algorithm (from the ‘sva’ package, v3.50.0)^12^, accounting for cancer type as covariates. To ensure data quality, we filtered out genes with very low expression, retaining only those with TPM > 1 in at least 10% of samples in the entire cohort. Given that batch effects in TCGA are often confounded with cancer type, we performed differential expression analyses for each cancer type independently. Comprehensive sensitivity analyses were performed to assess the impact of key preprocessing steps, specifically, we re-ran our entire differential expression analysis and tested three additional gene filtering thresholds, requiring at least 5, 20 and 30 counts in a subset of samples. The core findings, especially the differential expression of SPHK1 across various cancers, remained highly consistent under all tested conditions. SPHK1 expression was compared between tumor and normal tissues; the Wilcoxon rank-sum test was applied for unpaired comparisons across 33 TCGA cancer types, and the Wilcoxon signed-rank test was applied for paired comparisons within the same patient. All comparisons are visualized using box plots for clear presentation. Subsequently, a p-value threshold of less than 0.05 was set to indicate statistical significance. Additionally, we sourced immunohistochemical (IHC) images of proteins from the Human Protein Atlas (HPA) (accessible at https://www.proteinatlas.org/) to analyze SPHK1 expression in specific tumors and adjacent normal tissues.

### Diagnostic, prognostic value of SPHK1 in different types of cancer

The “pROC” (v1.18.5) package was used to create Receiver Operating Characteristic (ROC) curves for assessing diagnostic significance. Additionally, Cox regression analysis was performed to evaluate mortality risk, and Kaplan–Meier techniques were also performed for survival analysis. The prognostic significance of SPHK1 was investigated through the analysis of three metrics: Overall Survival (OS), Disease-Specific Survival (DSS), and Progression-Free Interval (PFI). PFI provides a comprehensive assessment of clinical outcome by capturing both disease progression and death, thereby reflecting the overall aggressiveness of tumors with SPHK1 dysregulation. This endpoint was considered more suitable than Disease-Free Interval (DFI) for our pan-cancer analysis, because it is less affected by heterogeneity in follow-up protocols and remains clinically relevant across all disease stages, especially in advanced cancers. For multivariate Cox regression, we consistently included age, pathological stage, and tumor grade as covariates across all cancer types. Missing clinical data were handled by complete-case analysis, and proportionality assumptions were verified using Schoenfeld residuals. These analyses were conducted using the “survival” (v3.3.1), “survminer” (v0.4.9) and “ggplot2” (v3.4.4) packages.

### Genetic alteration analysis

The cBioPortal platform (available at https://www.cbioportal.org/) was used to analyze the genetic modifications related to SPHK1 across different types of cancer. The alteration frequency of SPHK1 was obtained from the “Quick select” section of the “TCGA Pan Cancer Atlas Studies”. Data on mutations, amplifications, deep deletions, and other alterations were documented in the “Cancer Types Summary” module, which includes all tumors analyzed by TCGA. This analysis provided important information about the characteristics of the identified mutations, their occurrence frequencies, their specific locations, and the three-dimensional (3D) structure of the most commonly detected mutations.

### Analysis of SPHK1 gene expression levels and genomic heterogeneity

We used the R software package “TCGAplot” (v8.0.0) to investigate how SPHK1 expression levels correlate with tumor mutation burden (TMB) and microsatellite instability (MSI) across various cancer types from the Cancer Genome Atlas (TCGA)^13^.

### Protein–protein interaction network

We used the BioGRID (https://thebiogrid.org/) and STRING (https://cn.string-db.org/) databases to investigate potential protein–protein interaction networks**.** On the STRING platform, we entered “SPHK1” as the protein of interest and selected “Homo sapiens” as the organism. We chose “Experiments” as the main source of active interactions and limited the number of interactors to a maximum of 50. Additionally, we established a minimum interaction score of 0.150 to refine our results.

We used the “VennDiagram” (v1.7.3) and “ggplot2” (v3.4.4) packages in R to explore the overlaps between genes linked to SPHK1 and its interacting proteins. Additionally, we used the “ClusterProfiler” (v4.4.4) and “ggplot2” (v3.4.4) packages in R to conduct enrichment analyses on Gene Ontology (GO) and Kyoto Encyclopedia of Genes and Genomes (KEGG) pathways^14,15^.

### Co-expression of SPHK1 with immune cells and immune-related genes

We used spearman correlation analysis and Wilcoxon rank-sum tests to examine the relationships between pan-cancers and 22 different types of immune cells. Next, we performed another spearman correlation analysis to assess how SPHK1 co-expressed with various immune-related genes. This analysis included genes associated with immune checkpoints, chemokines, chemokine receptors, immune-stimulating factors, immunosuppressive components, and the overall immune score. The results are shown in a heat map, where the color intensity indicates the strength of the correlations; darker colors represent stronger relationships. For this analysis, we used the R software packages “ggplot2” (v3.4.4), “GSVA” (v1.46.0) and “pheatmap”(v1.0.12) to facilitate our data visualization and statistical analysis.

### Gene set enrichment analysis (GSEA)

To elucidate the signaling pathways associated with SPHK1 expression, we conducted a systematic Gene Set Enrichment Analysis (GSEA) focusing on three cancer types (HNSC, STAD, LIHC) which exhibited significant upregulation of SPHK1 at both mRNA and protein levels. Specifically, samples were first stratified into high- and low-SPHK1 expression groups using the median expression level of SPHK1 in tumor tissues as the cutoff. Subsequently, all samples were ranked based on fold change in gene expression. We then performed genome-wide KEGG pathway enrichment analysis using the “ClusterProfiler” R package (v4.4.4). Applying significance thresholds (*p* < 0.05 and FDR < 0.25), we ultimately identified five core signaling pathways that demonstrated the most pronounced enrichment.

### Drug sensitivity prediction

To identify potential therapeutic agents targeting SPHK1, we employed the CellMiner database (https://www.nci.nih.gov/cellminer) to systematically screen FDA-approved drugs and investigational compounds in clinical trials. To this end, SPHK1 mRNA expression data and the corresponding half-maximal inhibitory concentration (IC50) data were retrieved from CellMiner for the relevant cell lines. Subsequently, to validate the association between SPHK1 expression and drug sensitivity across multiple pharmacological databases, we performed Spearman correlation analyses using the GDSC and CTRP databases via the GSCA online platform (https://guolab.wchscu.cn/GSCA/#/drug). These analyses specifically assessed the relationships between SPHK1 expression levels and the IC50 values of selected small-molecule drugs.

### Real-time quantitative polymerase chain reaction

The tissue samples isolated from surgically excised tumor tissues and corresponding normal ones were frozen in liquid nitrogen within 2 h. Total RNA was extracted using PureLink® RNA Mini Kit (Invitrogen) according to the manufacturer’s guidelines. To remove potential DNA contaminants, the extracted RNA was treated with RNase-free DNase. The concentration and purity of nucleic acids were measured using the NanoDrop One (Thermo Fisher Scientific). OD260/280 ratios of the isolated RNA range from 1.80 to 2.00, indicating that the purity meets established standards. Agarose gel electrophoresis analysis of the isolated RNA revealed that the intensity of the 28S rRNA band was 1.5 to 2 times greater than that of the 18S rRNA band, and the RIN ≥ 7.0. Afterwards, 2 µg of RNA was used for synthesizing complementary DNA (cDNA) with random hexamer primers (Promega). Spike-in of an exogenous internal standard was used to detect potential inhibition. The reverse transcription process involved heating 2 μg of total RNA and 2 μl of Oligo(dT) at 70 °C for 5 min, then quickly cooling it on ice. This mixture was then combined with a reaction solution containing 5 μl of M-MLV RT 5X Buffer (Promega, USA), 0.5 μl of 25 μM deoxyribonucleotide triphosphate (Promega, USA), 0.7 μl of RNase inhibitor (Promega, USA), and 1 μl of M-MLV reverse transcriptase (Promega, USA). The total reaction volume was adjusted to 25 μl with RNA-free water. It was then incubated at 42 °C for 1 h, followed by a 10-min incubation at 70 °C. The synthesized cDNA was then stored at -80 °C until use. The sequence accession number for SPHK1 is NM_001142602.2, transcript variant 4, the primers used in this study included SPHK1 forward primer (5 ‘- TCAGTCTGTCCTGGGGTTTC-3’), SPHK1 reverse primer (5 ‘- TCCTCCAGAGGAACGAGGTA-3’). The product size (BLAST) is 229 bp. In the RT-PCR procedure, QuantiTect SYBR Green PCR Kit (QIAGEN, USA) was used according to the manufacturer’s instructions on Cobas Z480 (Roche, Switzerland), we prepared a reaction mixture consisting of 5 μl of QuantiTect SYBR Green PCR Master Mix, 0.5 μl of 10 μM forward primer, 0.5 μl of 10 μM reverse primer, cDNA template, and RNAse-free water. The PCR amplification was conducted under a series of specific conditions: an initial denaturation step at 95 °C for 15 min, followed by 45 cycles including denaturation at 94 °C for 15 s, annealing at 61 °C for 22 s, and extension at 72 °C for 30 s. The melting curve shows a single peak, the standard curve exhibits a slope of − 3.3 with R^2^ = 0.996, and the efficiency is 100.8%. The expression data were measured in triplicate and normalized against the housekeeping gene GAPDH, which served as a loading control. To explore potential correlations, we used either Spearman’s rank correlation or Pearson correlation analysis, depending on the data characteristics. All statistical calculations and visualizations were performed with R software (Version 4.4).

#### Cell culture, transfection and cell viability assay

SCC-25, MGC-803 and HepG2 cell lines were purchased from the Institute of Biochemistry and Cell Biology at the Chinese Academy of Sciences (Shanghai, China). The three cell lines were confirmed through (Short Tandem Repeat, STR) typing analysis, and all tests for mycoplasma, bacteria, and fungi were negative. Upon cultivation, the observed cellular morphologies were consistent with the characteristic morphologies of these three cell types. Then, SCC-25, MGC-803 and HepG2 cell lines were cultured in DMEM supplemented with 10% fetal bovine serum and penicillin–streptomycin. The lentivirus for SPHK1 knockdown was sourced from OBiO Technology located in Shanghai, China. The CCK-8 assay was used to evaluate how SPHK1 knockdown affects the proliferation of HNSC, STAD and LIHC cells. cells were plated in 96-well plates at a density of 5000 cells per well. After a 24 h incubation at 37 °C, SPHK1 shRNA was added to the appropriate wells. Then, 10 µL of CCK-8 reagent (Beyotime Institute of Biotechnology, China) was added to each well at specified time intervals. The wells were then incubated for an additional 2 h. Finally, the absorbance was measured at a wavelength of 450 nm using a spectrophotometer.

#### Immunohistochemistry assay

Patients who underwent radical surgery between April 2018 and August 2023 at Sichuan Cancer Hospital & Institute were included in this study, and we collected tissue specimens from HNSC, STAD, and LIHC, with 20 samples designated for each type. The inclusion criteria were as follows: (1) histopathologically confirmed HNSC, STAD or LIHC; (2) patients could provide sufficient tissues to meet the requirements of the study; (3) patients were followed up and were willing to join the study. Exclusion criteria included: (1) presence of multiple primary tumors; (2) patients with follow-up loss within a month. (3) Patients who have received neoadjuvant therapy (preoperative radiotherapy and chemotherapy). Missing clinical data were handled by complete-case analysis, and proportionality assumptions were verified using Schoenfeld residuals. Liver cancer tissue with high SPHK1 expression confirmed by Western blot was used as positive control, while the negative control was performed by replacing the primary antibody with antibody dilution buffer, keeping all other steps unchanged. Specimens were sliced into 4 μm sections and then deparaffinized them using xylene after heat drying. To facilitate heat-induced epitope retrieval, we treated the sections with heated citrate buffer for 20 min. We inhibited endogenous peroxidase activity using a 3% hydrogen peroxide solution. Three pathologists scored the slides independently, and we used the average of their evaluations for further analysis. All procedures performed in this study involving human participants were in accordance with the Declaration of Helsinki (as revised in 2013). The study was approved by the ethics committee of Sichuan Cancer Hospital & Institute (Approval Number: SCCHEC-02-2019-016), and received informed consent from all participants.

#### Statistical analysis

We used the Kruskal–Wallis Test along with the Wilcoxon rank-sum test to assess differences in gene expression between normal and malignant tissues. For survival analysis, we conducted Kaplan–Meier analysis, supported by the Cox proportional hazards model and log-rank tests. To investigate potential correlations, we used either Spearman or Pearson correlation analysis, depending on the specific characteristics of the data, and all statistical computations and visual representations were performed using R software. And we defined the results as statistically significant at *p* < 0.05 (*), *p* < 0.01 (**), and *p* < 0.001 (***).

## Results

### The pan-cancer analysis of SPHK1 mRNA expression

**Fig. 1:**
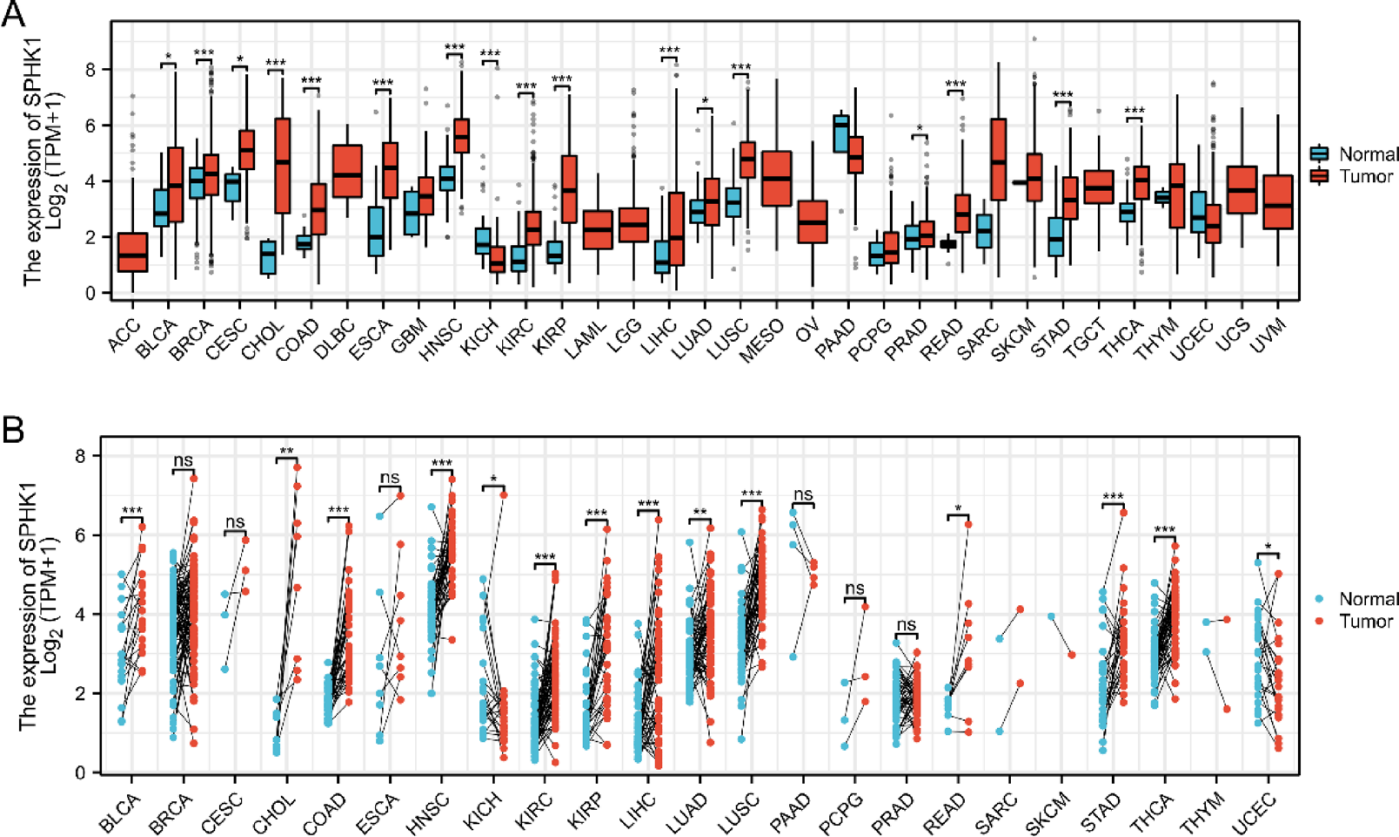
SPHK1 mRNA expression levels in different tumor types. (**A**) SPHK1 mRNA expression in different tumors VS normal controls. (**B**) SPHK1 mRNA expression in different tumors VS corresponding matched normal controls. *p < 0.05; **p < 0.01; ***p < 0.001.

SPHK1 mRNA expression levels were assessed in tumor and normal tissues from 33 different tumor types obtained from TCGA. The results showed that SPHK1 expression was significantly increased in 16 tumor types, while only one tumor type showed a decrease, as illustrated in Fig. 1A. Figure 1B shows the expression differences of SPHK1 between tumor tissues and their normal counterparts. SPHK1 was upregulated in 12 tumor types and downregulated in two. Overall, SPHK1 expression was increased in 12 tumor types in both matched and unmatched samples. These include bladder cancer (BLCA), cholangiocarcinoma (CHOL), colon adenocarcinoma (COAD), head and neck squamous cell carcinoma (HNSC), kidney renal clear cell carcinoma (KIRC), kidney renal papillary cell carcinoma (KIRP), liver hepatocellular carcinoma (LIHC), lung adenocarcinoma (LUAD), lung squamous cell carcinoma (LUSC), rectum adenocarcinoma (READ), stomach adenocarcinoma (STAD), and thyroid carcinoma (THCA), with all statistical comparisons showing P-values below 0.05. In contrast, only one tumor type, kidney chromophobe (KICH), showed a decrease in SPHK1 expression in both matched and unmatched samples.

We then analyzed the mRNA expression levels of SPHK1 across various pathological stages in a pan-cancer study. Significant differences in SPHK1 expression were found among nine different tumor types (Supplementary Fig. S1). For instance, in uterine endometrial carcinoma (UCEC), the expression levels were recorded as follows: Stage I—343 cases, Stage II—52 cases, Stage III—130 cases, and Stage IV—29 cases. In THCA, there were 288 cases in Stage I, 52 in Stage II, 113 in Stage III, and 57 in Stage IV. Finally, in adrenocortical carcinoma (ACC), there were 9 cases in Stage I, 37 in Stage II, 16 in Stage III, and 15 in Stage IV.

### Protein expression levels of SPHK1 between tumor and normal tissue samples

**Fig. 2:**
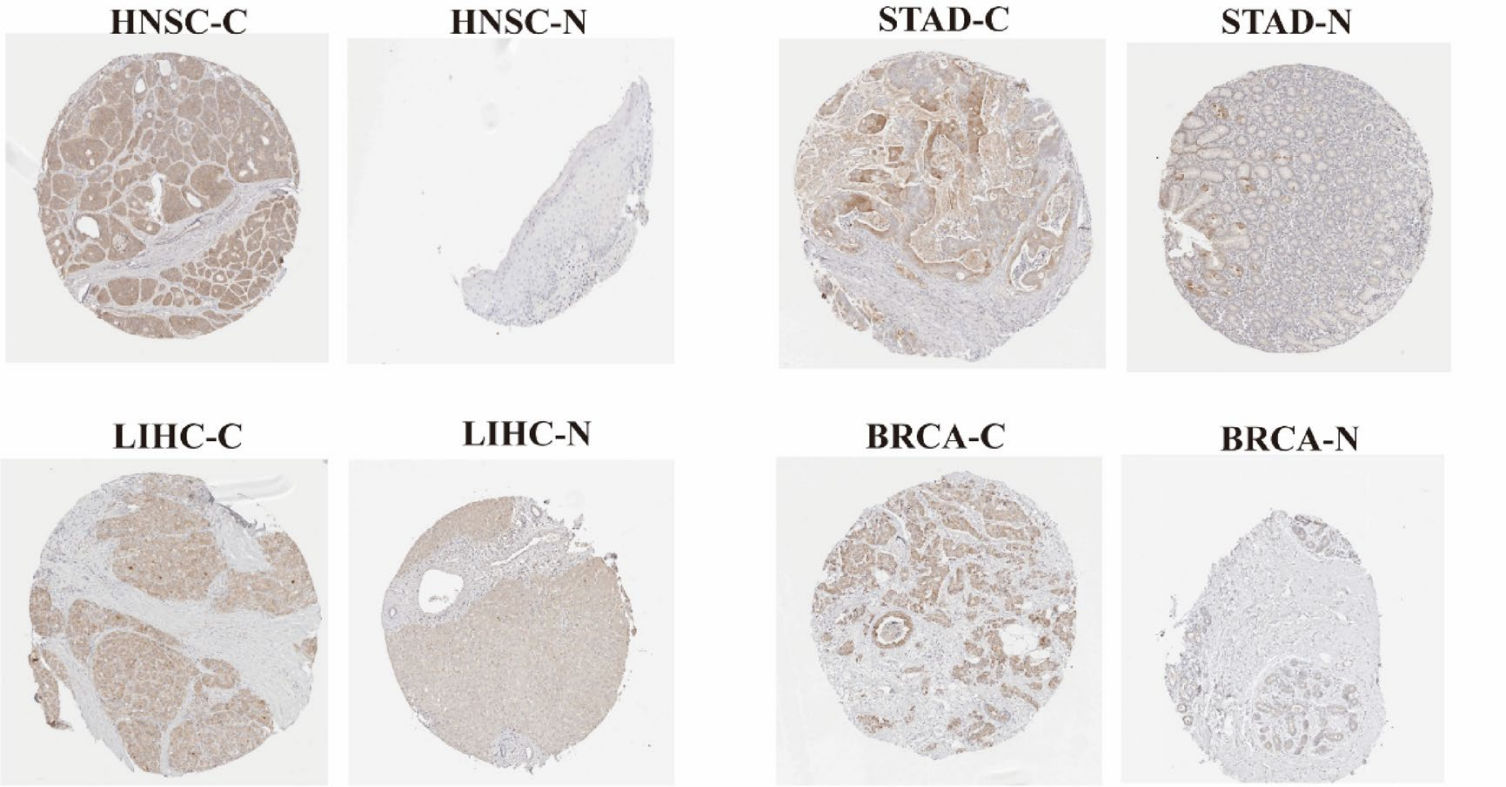
Protein expression of SPHK1 in different tumor samples and corresponding normal tissues. Protein expression of SPHK1 was significantly higher in HNSC, STAD, LIHC, and BRCA. C represents tumor tissue; N represents normal tissue.

The Human Protein Atlas (HPA) data analysis revealed that several cancers, HNSC, STAD, LIHC and BRCA displayed immunohistochemical (IHC) staining with moderate to high intensity. In contrast, normal tissues associated with these cancers showed little to no IHC staining. As was shown in Fig. 2.

### Diagnostic value of SPHK1 in pan-cancer

**Fig. 3:**
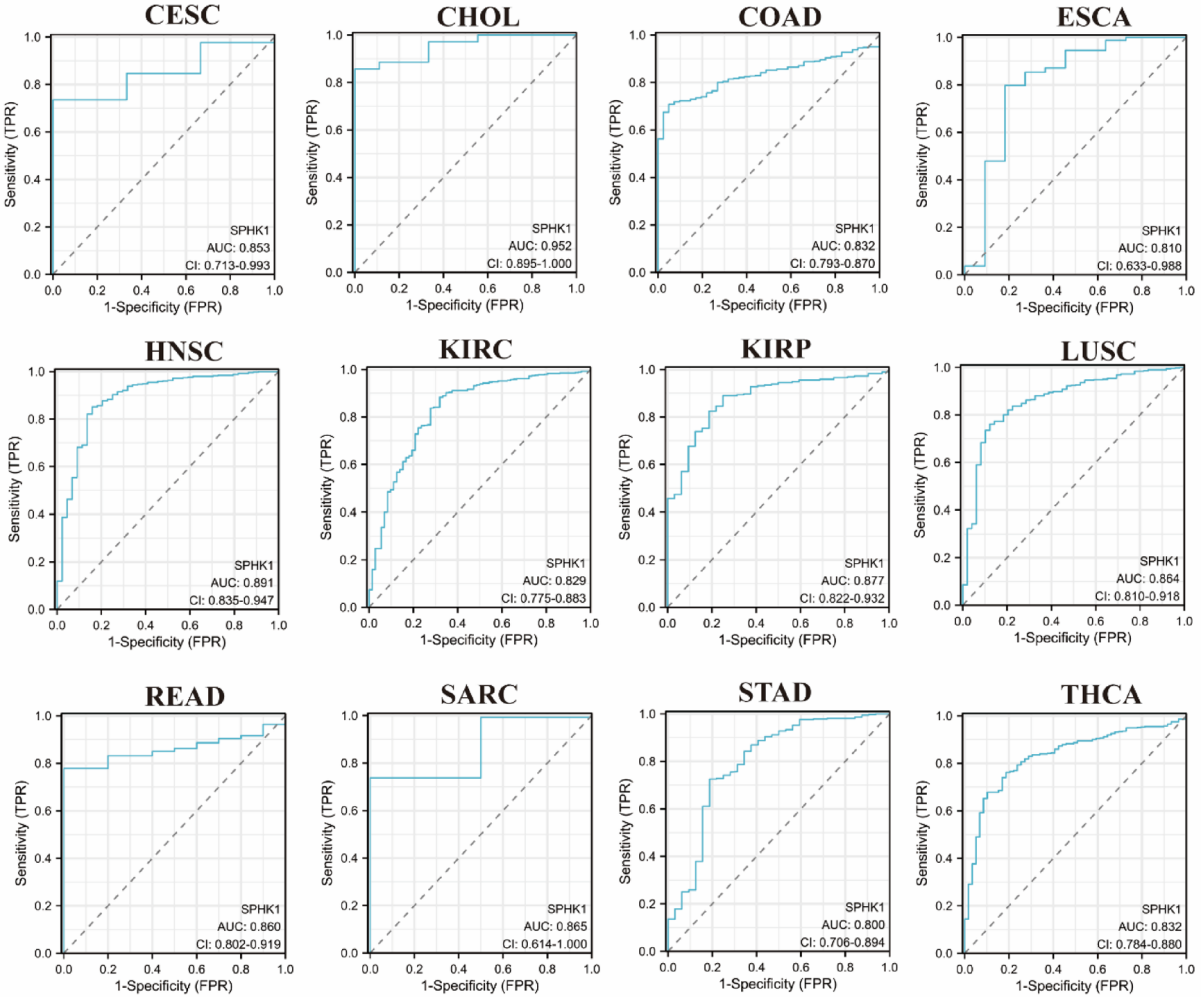
Receiver operating characteristic (ROC) curve for SPHK1 expression in pan-cancer.

The Receiver Operating Characteristic (ROC) curve was used to assess how well SPHK1 expression can differentiate individuals with various pan-cancer tumors from those without any tumors. The area under the curve (AUC) values for CHOL was 0.952, indicating SPHK1 expression is highly accurate in distinguishing CHOL from nearby normal tissues. Furthermore, the AUC values for Cervical squamous cell carcinoma and endocervical adenocarcinoma (CESC), HNSC, KIRP, LUSC, READ, sarcoma (SARC), COAD, esophageal carcinoma (ESCA), KIRC, STAD, and THCA, which were 0.853, 0.891, 0.877, 0.864, 0.860, 0.865, 0.832, 0.810, 0.829, 0.800, and 0.832, respectively, indicate a moderate level of accuracy for these tumors (Fig. 3).

### Prognostic value of SPHK1 in pan-cancer

**Fig. 4:**
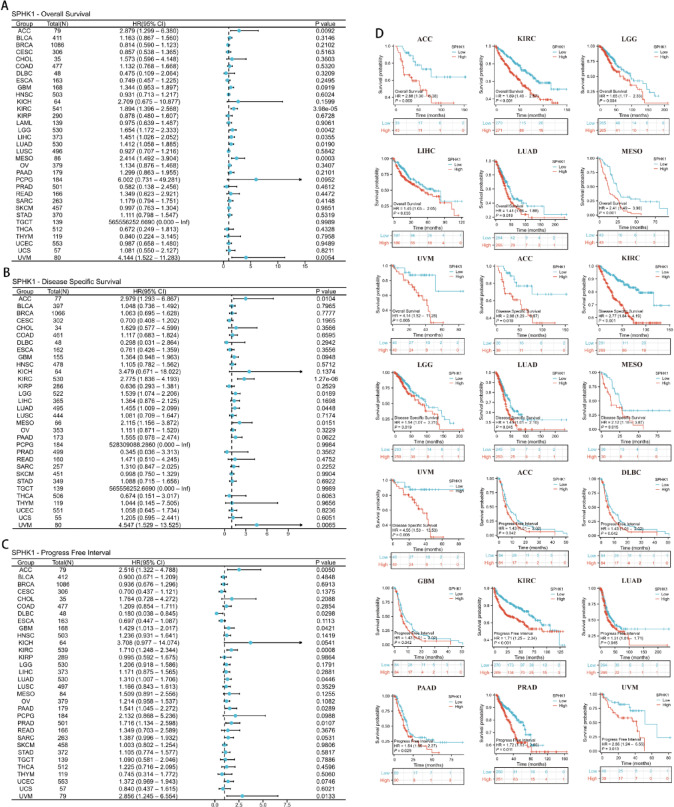
Correlation between SPHK1 mRNA expression and survival prognosis in pan-cancer. (**A**) Forest plots between SPHK1 and OS in pan-cancer; (**B**) Forest plots between SPHK1 and DSS in pan-cancer; (**C**) Forest plots between SPHK1 and PFI in pan-cancer; (**D**) Kaplan–Meier Curves of ACC, KIRC, LGG, LIHC, LUAD, MESO, UVM, DLBC, GBM, PAAD and PRAD.

The univariate Cox regression analysis revealed a significant link between high SPHK1 expression levels and reduced overall survival (OS) across several cancer types, including ACC, KIRC, LGG, LIHC, LUAD, mesothelioma (MESO), and uveal melanoma (UVM) (see Fig. 4A and D). Furthermore, forest plots and Kaplan–Meier survival analyses confirmed the association between elevated SPHK1 expression and lower disease-specific survival (DSS) in ACC, KIRC, brain lower grade glioma (LGG), LUAD, MESO, and UVM (illustrated in Fig. 4B and D). Additionally, increased SPHK1 expression was linked to a poorer progression-free interval (PFI) in ACC, lymphoid neoplasm diffuse large B-cell lymphoma (DLBC), glioblastoma multiforme (GBM), KIRC, LUAD, pancreatic adenocarcinoma (PAAD), prostate adenocarcinoma (PRAD), and UVM, as shown in Fig. 4C and D. To verify whether SPHK1 is an independent prognostic factor for tumors, we conducted a multivariate cox proportional hazards regression analysis on key clinical prognostic factors (such as age, tumor stage, and tumor grade) and the SPHK1 expression level. It was found that SPHK1 remained an independent factor for OS in ACC, KIRC, LIHC, MESO and uveal UVM (Supplementary Tables S1–S5). Additionally, SPHK1 was an independent prognostic factor for DSS in ACC, KIRC, MESO, and UVM (Supplementary Tables S6–S9). Furthermore, it was an independent prognostic factor for PFI in DLBC and UVM (Supplementary Tables S10–S11).

### Genetic alteration of SPHK1 in pan-cancer

The cBioPortal platform integrates data from TCGA to investigate genetic modifications of the SPHK1 gene in various cancer types. Skin cutaneous melanoma (SKCM) had the highest prevalence of gene alterations at 4.95%. Amplification was the most common type of alteration across cancers, likely increasing SPHK1 expression. Supplementary Fig. S3A shows that breast invasive carcinoma (BRCA) had the highest frequency of amplification at 3.69%. In contrast, SKCM had the highest mutation frequency at 2.48%. The R191H mutation was the most frequently detected (see Supplementary Fig. S2C), with missense and truncating mutations being the main types affecting SPHK1. Supplementary Fig. S2B shows the 3D layout of the mutated site.

### Analysis of SPHK1 expression levels correlated with gene heterogeneity

**Fig. 5:**
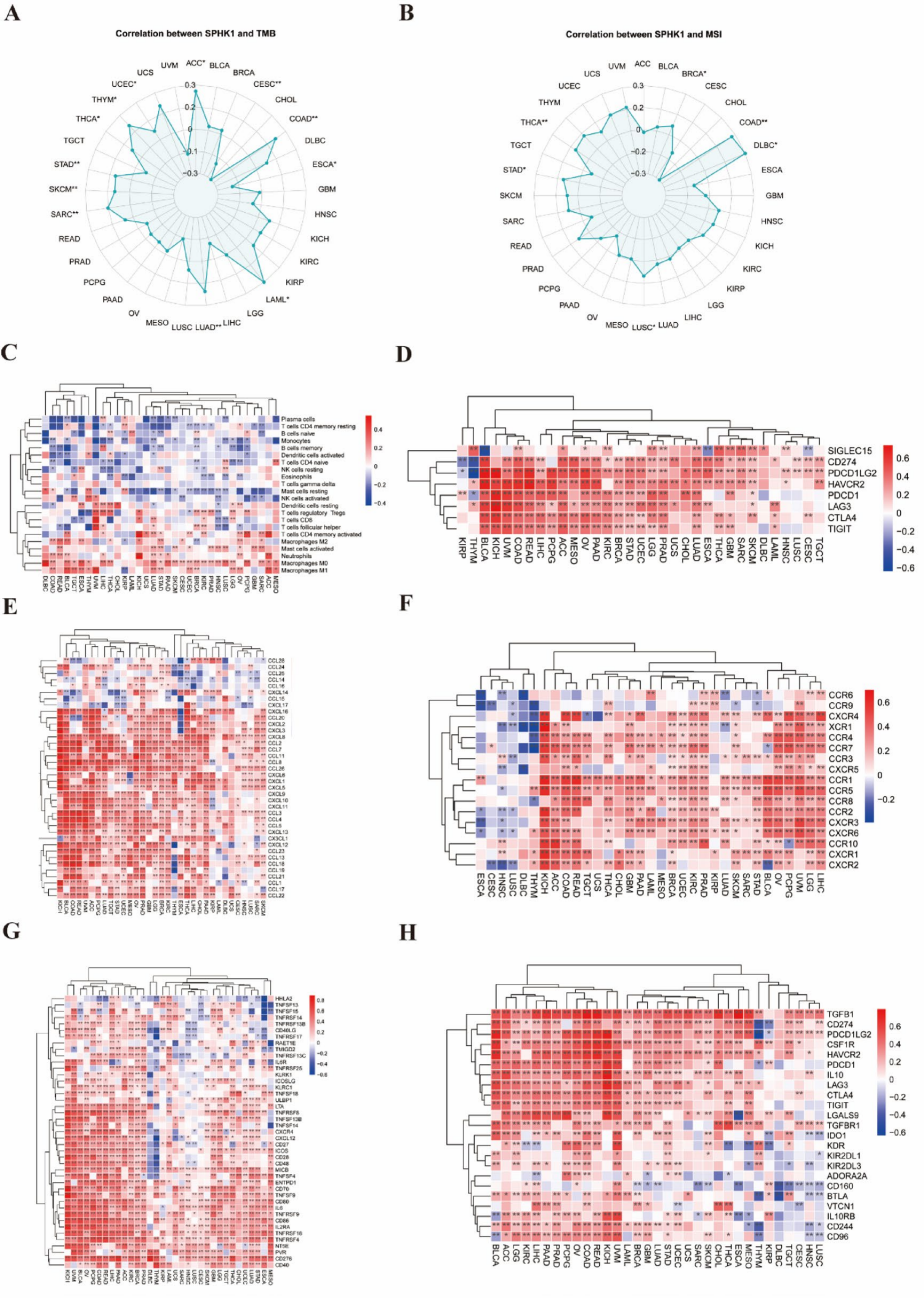
Correlation between SPHK1 and gene heterogeneity, and coexpression of SPHK1 with immune cells and immune-related genes. (**A**). Correlation between expression of SPHK1 and TMB. (**B**) Correlation between expression of SPHK1 and MSI. *p < 0.05; **p < 0.01. (**C**) Coexpression of SPHK1 with immune cells. (**D**) Coexpression of SPHK1 with checkpoint-related genes; (**E**) Coexpression of SPHK1 with chemokine-related genes; (**F**) Coexpression of SPHK1 with chemokine receptor-related genes; (**G**) Coexpression of SPHK1 with immune activation genes; (**H**) Coexpression of SPHK1 with immunosuppressive genes. *p < 0.05; **p < 0.01.

Figure 5A shows a significant positive correlation between TMB and SPHK1 expression across ten tumor types: ACC, COAD, acute myeloid leukemia (LAML), LUAD, SARC, SKCM, STAD, THCA, thymoma (THYM), and UCEC, all with P-values below 0.05. In contrast, significant negative correlations were found in CESC and ESCA, both exhibiting P-values below 0.05. Furthermore, we examined the relationship between MSI and various cancers in TCGA. The results showed a significant positive correlation in six tumor types: BRCA, COAD, DLBC, LUSC, STAD, and THCA, all with P-values below 0.05. Notably, no tumor types showed a negative correlation between SPHK1 expression and MSI, as seen in Fig. 5B.

### Co-expression of SPHK1 with immune cells and immune-related genes

We began by analyzing the correlation between SPHK1 expression levels and 22 different types of immune cells. Figure 5C demonstrates a positive association between SPHK1 expression and M_0_ macrophages in almost all cancer types in the TCGA dataset. Next, we examined the co-expression patterns of SPHK1 with various immune-related genes across different cancer types. This analysis included genes related to checkpoints, chemokines, chemokine receptors, immune stimulation, and immunosuppression, as well as immune scoring metrics, as shown in Fig. 5D–H.

Figure 5D shows a strong positive relationship between the checkpoint-related genes HAVCR2, LAG3, and CTLA4, along with SPHK1, across various cancer types studied in TCGA. Additionally, chemokine-related genes like CXCL8, CCL2, and CCL8 show a positive correlation with many cancers (Fig. 5E). Figure 5F reveals a strong association between the expression of several genes related to chemokine receptors, especially in KICH, LIHC, and LGG. Figure 5G shows a robust connection between the expression of immune-stimulating genes, including TNFRSF18, TNFRSF4, and CD276, across nearly all cancer types. In terms of immunosuppressive genes, Fig. 5H shows that SPHK1 has a notable correlation with TGFB1 in most cancer types, except for DLBC and TGCT.

### The protein–protein interaction network of SPHK1 in pan-cancer

**Fig. 6:**
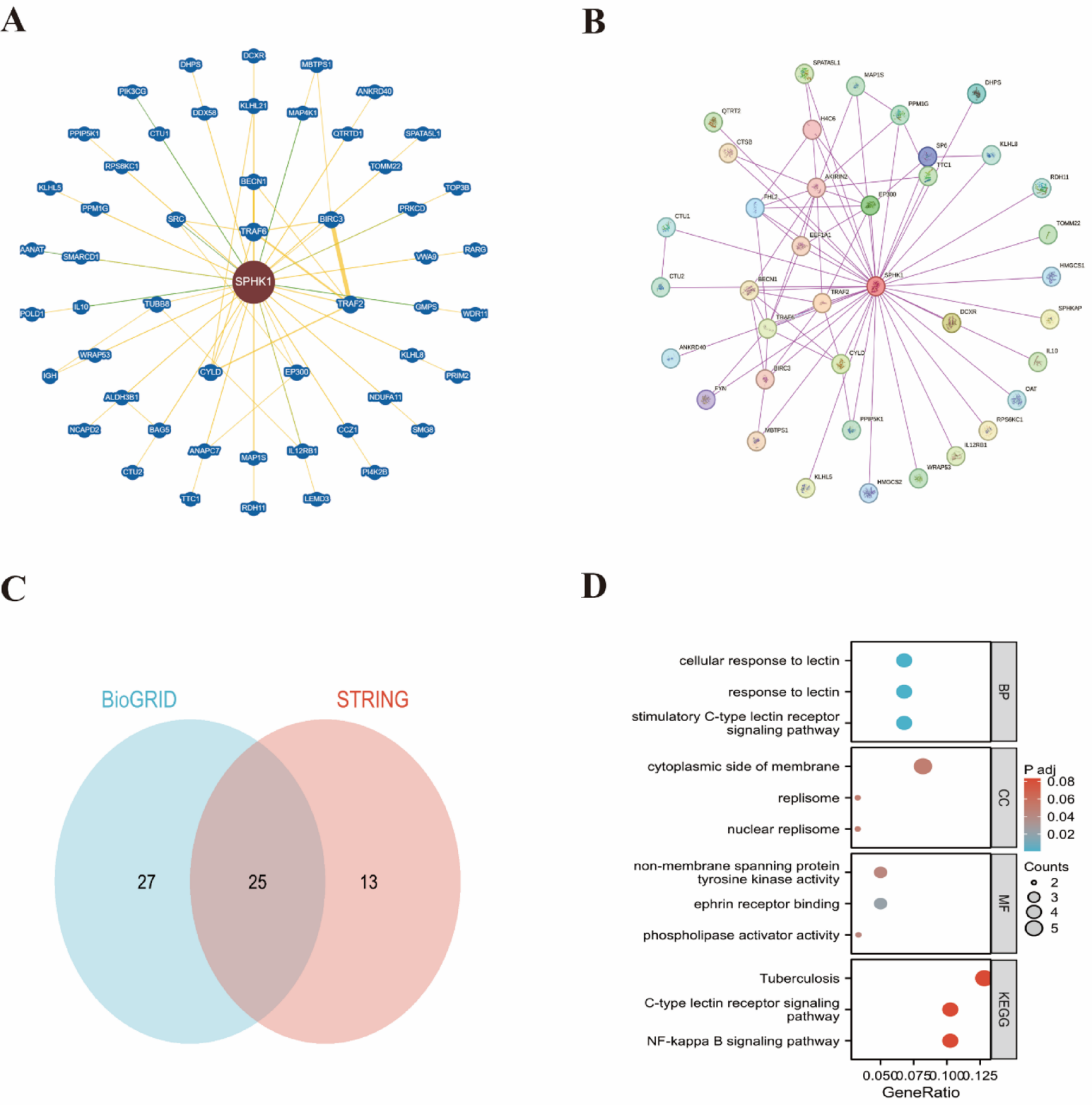
The Protein–protein interaction analysis of SPHK1. (**A**) 52 SPHK1-related genes were obtained from the BioGRID website. (**B**) A total of 38 proteins that bind to SPHK1 were identified from the STRING tool. (**C**) The intersection graph of the SPHK1-correlated and SPHK1-binding genes. (**D**) The GO and KEGG analysis of the SPHK1-binding and interacting genes.

Genes that may be linked to SPHK1 were obtained from the BioGRID database, identifying approximately 52 genes (see Fig. 6A). A search in the STRING database revealed about 38 proteins that interact with SPHK1 (see Fig. 6B). Notably, 25 genes were identified that overlap between the SPHK1-associated genes and the interacting proteins (illustrated in Fig. 6C). To further investigate these genes, we performed GO and KEGG enrichment analyses on the combined set of genes related to or interacting with SPHK1^16^, using the “clusterProfiler” package. The three pathways most strongly correlated with SPHK1 were tuberculosis, the C-type lectin receptor signaling pathway, and the NF-kappa B signaling pathway (shown in Fig. 6D).

### The enrichment signaling pathways of SPHK1 in pan-cancer

An enrichment analysis was conducted on 3 tumor types that exhibited notable up-regulation of SPHK1 in both mRNA and protein levels. Supplementary Fig. S3A illustrates the five most significantly enriched up-regulated pathways for HNSC, which include “Cytoskeleton in muscle cells”, “Dilated cardiomyopathy”, “ECM − receptor interaction”, “Rheumatoid arthritis”, and “Protein digestion and absorption”. For STAD, the analysis identified the top three enriched up-regulated pathways: “ECM − receptor interaction”, “Cytoskeleton in muscle cells”, and “Phagosome”. Conversely, two pathways were observed to be down-regulated, specifically “Metabolism of xenobiotics by cytochrome P450” and “Drug metabolism − cytochrome P450”, as shown in Supplementary Fig. S3B. and Supplementary Fig. S3C presents the five most significantly enriched down-regulated pathways for LIHC: “Chemical carcinogenesis − DNA adducts”, “Drug metabolism − cytochrome P450”, “Metabolism of xenobiotics by cytochrome P450”, “Retinol metabolism”, and “Steroid hormone biosynthesis”.

#### The correlation of SPHK1 with drug sensitivity in pan-cancer

**Fig. 7:**
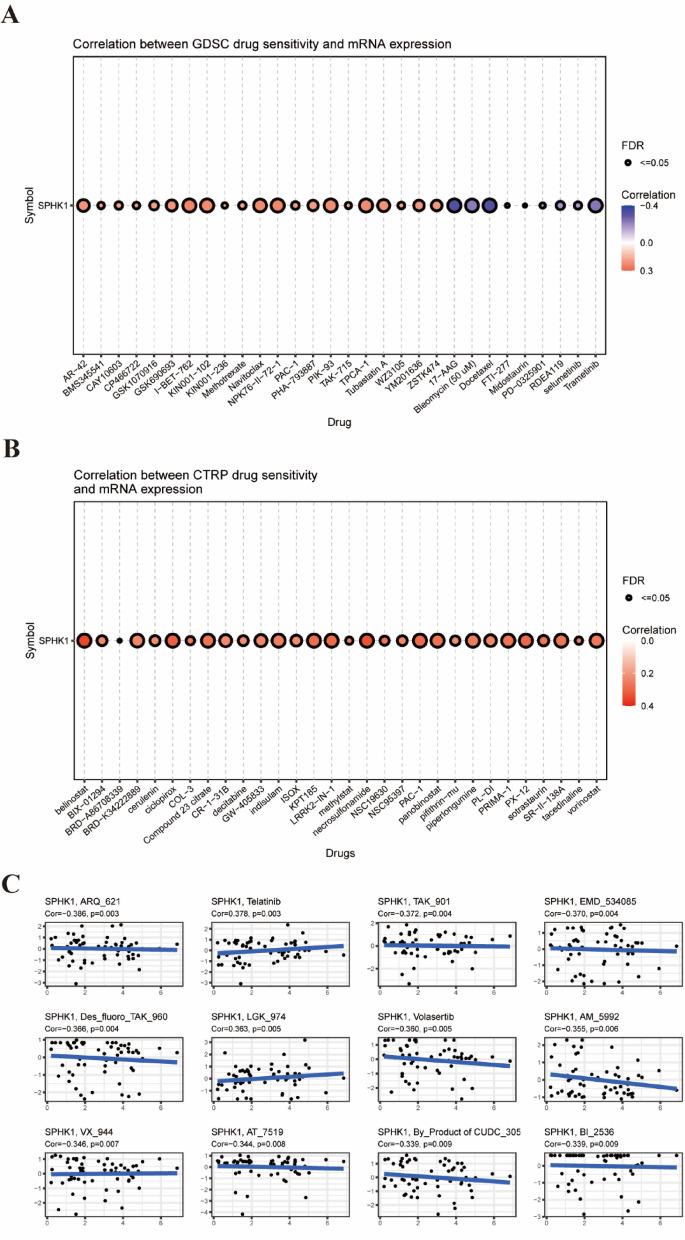
The association of SPHK1 with drug sensitivity in pan-cancer. (**A**) Drugs that were significantly associated with expression of SPHK1 based on GDSC database. (**B**) Drugs that were significantly associated with expression of SPHK1 based on CTRP database (**C**) Drugs that were significantly associated with expression of SPHK1 based on CellMiner database.

Our analysis identified a significant link between SPHK1 expression levels and drug sensitivity across various cancer types, based on data from the GDSC, CTRP, and CellMiner databases. In the GDSC dataset, SPHK1 expression positively correlated with the IC50 values of several pharmacological agents, such as Methotrexate, Navitoclax, and others. Conversely, a negative correlation was noted with the IC50 values of agents like 17 − AAG, Bleomycin (50 μM), and others (Fig. 7A). In the CTRP database, SPHK1 expression was positively correlated with the IC50 values of various agents, including belinostat, BIX − 01,294, and vorinostat (Fig. 7B). The CellMiner database reveals a positive correlation between SPHK1 expression and the activity of compounds like Telatinib (R = 0.378, P = 0.003) and LGK_974 (R = 0.363, P = 0.005). Conversely, a negative correlation was found with the activity of several compounds, including ARK_621 (R = -0.386, P = 0.003) and TAK_901 (R = -0.370, P = 0.004), among others (Fig. 7C). These findings suggest that SPHK1 may play a potential role in cancer treatment and that targeting SPHK1 with anti-cancer therapies may enhance their effectiveness.

#### Expression of SPHK1 in surgical resected HNSC, STAD and LIHC tissues.

**Fig. 8:**
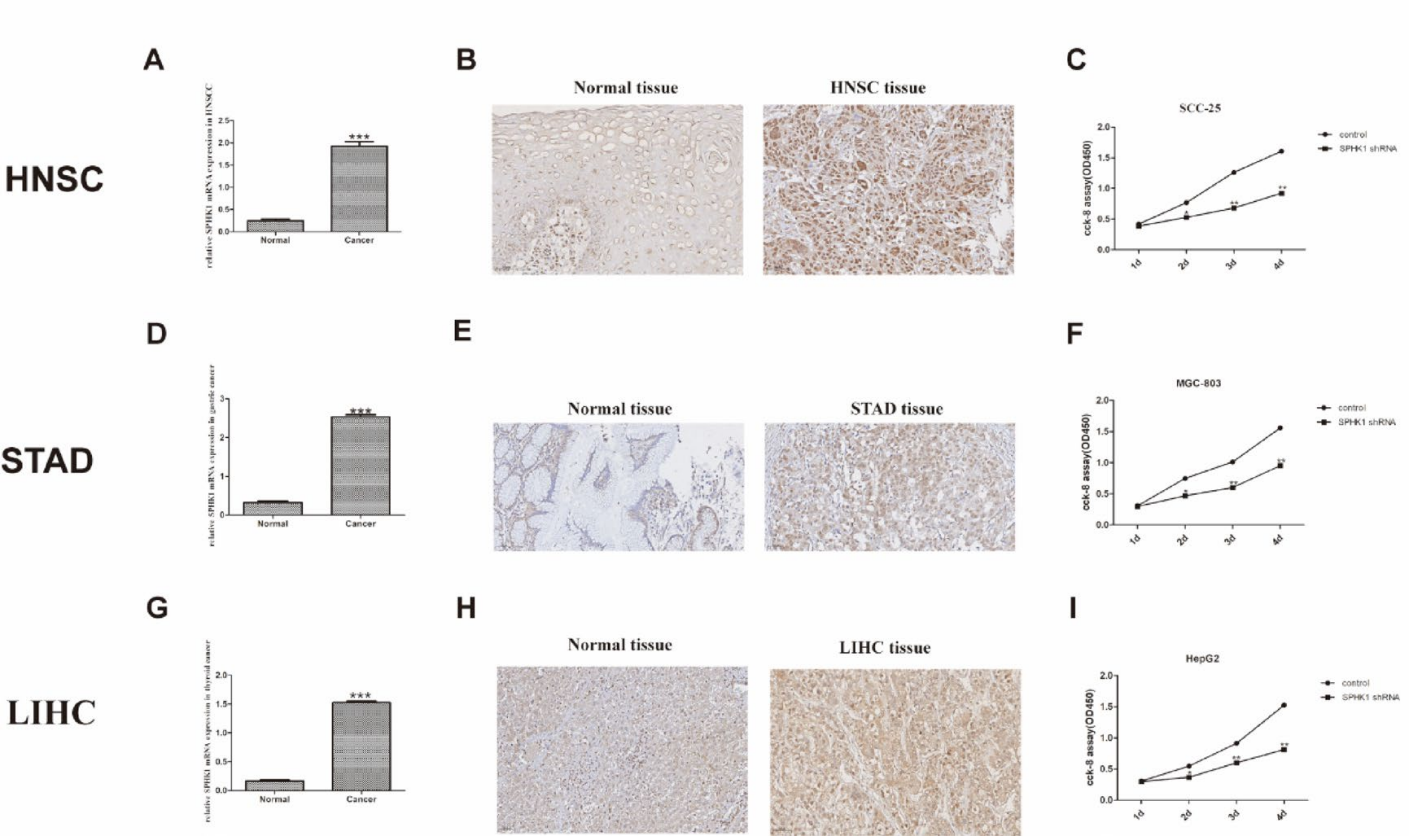
Relative mRNA and protein expression of SPHK1 in HNSC, STAD and LIHC samples and the growth function of SPHK1 in HNSC, STAD and LIHC cell lines. SPHK1 mRNA was up-regulated in HNSC (**A**), STAD (**D**) and LIHC (**G**) cancer tissues compared with adjacent normal tissues. SPHK1 protein was up-regulated in HNSC (**B**), STAD (**E**) and LIHC (**H**) cancer tissues compared with adjacent normal tissues. SPHK1 promotes cellular proliferation of SCC-25 (**C**), MGC-803 (**F**) and HepG2 (**I**) cells. *p < 0.05; **p < 0.01; ***p < 0.001.

To validate the actual expression levels of SPHK1 in clinical samples, we selected cancer types with both mRNA and protein levels of SPHK1 significantly higher in tumors than in normal tissues for further validation, including 20 pairs of HNSC, STAD and LIHC tissues and corresponding normal ones separately. Real-time quantitative PCR and immunohistochemistry analysis on surgical specimens from patients with HNSC, STAD and LIHC were used to assess the expression levels of SPHK1. Normal adjacent tissues were used as controls for comparison. The clinicopathological characteristics of the three types of tumors are presented in Supplementary Table S12. The results showed a significant increase in SPHK1 mRNA and protein expression in HNSC STAD and LIHC tissues compared to normal tissues, with a p-value of less than 0.001, indicating statistical significance (see Fig. 8A, B, D, E, G, H). Kaplan–Meier survival analysis was performed for three cancer types (HNSC, STAD, LIHC). The results, shown in Supplementary Fig. S4A–C, indicate that high SPHK1 protein expression is significantly associated with poorer overall survival in these cancers (Cox regression P = 0.04, P = 0.035, and P = 0.038, respectively). We quantified the transcript-protein concordance in three validated cancer types. The results show a significant positive correlation between SPHK1 mRNA and protein levels in HNSC (Spearman R = 0.483, P = 0.004), STAD (R = 0.542, *p* < 0.001), and LIHC (R = 0.42, P = 0.013). See Supplementary Fig. S4D–F for details. We also analyzed SPHK1 expression in HNSC (GSE107591), STAD (GSE26942), and LIHC (GSE57957) datasets from the GEO database, and found that its levels were higher in tumor tissues than in adjacent or normal tissues (supplementary Fig. S5).

#### SPHK1 promotes viability of HNSC, STAD and LIHC cells

The CCK-8 assay was used to investigate the viability potential of HNSC, STAD and LIHC cells. SCC-25 cells treated with SPHK1 shRNA showed significantly reduced viability compared to the control group (Fig. 8C). Similarly, MGC-803 and HepG2 cells treated with SPHK1 shRNA exhibited significant inhibition of viability, also demonstrating a statistically significant difference (Fig. 8F and I). These findings confirm that reducing SPHK1 expression levels hinders tumor viability in vitro.

## Discussion

Cancer, a major global health challenge, requires research that overcomes the limitations of focusing on individual cancer types because of its heterogeneity and complexity^17^. Pan-cancer analysis, by systematically comparing similarities and differences among tumors, thus provides critical insights into universal biological principles and the development of broad-spectrum therapeutic strategies.^18^. This study focuses on SPHK1, the key rate-limiting enzyme in the sphingolipid metabolism pathway. Notably, SPHK1 is widely defined as an oncogene due to its role in promoting cell proliferation, survival, and chemotherapy resistance by catalyzing the production of S1P.^19,20^. However, recent research has clearly revealed the close link between tumor metabolic reprogramming and immune evasion, in which specific metabolic enzymes actively shape an immunosuppressive tumor microenvironment, functioning like “metabolic immune checkpoints”^21^. This study conducts a systematic pan-cancer analysis and, for the first time, places SPHK1 within a “metabolism-immunity” interaction framework. The aim is to comprehensively redefine SPHK1’s role from a classic cancer-promoting factor to a key hub connecting intrinsic metabolic dysregulation in tumor cells with external immunosuppressive states.

Our analysis revealed that SPHK1 is overexpressed in various cancers, with an area AUC of 0.800 or higher in 12 tumor types, such as CESC, CHOL, and HNSC, indicating its potential as a diagnostic biomarker. Our bioinformatics analysis suggests a correlation between high SPHK1 expression and poor prognosis in multiple cancers, including OS, DSS, and PFI. This correlation may imply that SPHK1 has potential as a prognostic biomarker. Additionally, multivariate Cox regression analysis supports its role as a potential independent prognostic risk factor. Although these retrospective findings based on public databases require validation through prospective cohorts, they have preliminarily established the clinical translational value of SPHK1. We further emphasize that the prognostic significance of SPHK1 may not only result from its direct proliferative effects. It is also likely linked to its core functions in remodeling the immune microenvironment and promoting immunosuppression. To thoroughly elucidate its underlying mechanisms, we performed a multidimensional integrative analysis. This approach allowed us to explore various related pathways comprehensively. GSEA identified that SPHK1-associated genes were significantly enriched in pathways related to extracellular matrix receptor interaction, drug metabolism, and immune regulation, suggesting its extensive involvement in the construction and functional regulation of the tumor microenvironment^22,23^. A key breakthrough emerged from the immune landscape analysis: SPHK1 expression was positively correlated with TMB and MSI, implying that tumors with high SPHK1 expression may have a higher baseline of immunogenicity. Our pan-cancer analysis, for the first time, systematically revealed that SPHK1 may be closely linked to immune evasion. First, SPHK1 shows significant positive correlations with key immune checkpoints HAVCR2 (TIM-3), LAG3, and CTLA4, indicating that in tumors with high SPHK1 expression, T cells are functionally exhausted and unable to effectively kill tumor cells. Second, SPHK1 is positively correlated with chemokines that recruit immunosuppressive cells, suggesting that SPHK1 may create an immune-suppressive environment by mobilizing these ‘suppressive forces,’ which helps tumors evade immune attacks. Third, SPHK1 and TGFB1 are co-expressed in the vast majority of cancer types, providing a direct and potent molecular pathway through which it may promote immune evasion. This perfectly aligns with the definition of a “metabolic immune checkpoint,” which is defined as a critical node that actively suppresses anti-tumor immune responses through metabolic reprogramming.

This study constructed, for the first time, a multidimensional regulatory map of SPHK1 across cancers, surpassing the focus on its tumor-promoting role in a single cancer. This new positioning holds profound translational significance: Firstly, co-expression of SPHK1 with multiple immune checkpoints suggests that targeting SPHK1 together with PD-1/CTLA-4 pathways could produce synergistic effects, reversing immunotherapy resistance^24^. Secondly, drug sensitivity analysis shows that high SPHK1 expression correlates with sensitivity to the BCL-2 inhibitor navitoclax, providing clues for precision treatment stratification based on SPHK1 expression. Targeting the SPHK1/S1P axis has emerged as a promising strategy that may simultaneously inhibit tumor growth and reverse immunosuppression^7^.

Previous studies have mostly focused on SPHK1-mediated chemotherapy resistance in response to complex drug sensitivity associations^25,26^. However, our pan-cancer analysis revealed a bidirectional regulatory pattern of SPHK1 on drug efficacy. This seemingly contradictory phenomenon may stem from several factors. First, the bidirectionality of the SPHK1/S1P signaling pathway. S1P can generate diametrically opposed cellular fate signals through different receptors (e.g., S1PR1 promotes survival^27^, whereas S1PR2 and S1PR5 induce apoptosis)^28,29^, and the net effect depends on the cellular context and the mechanism of drug action. Second, SPHK1 may weaken the efficacy of platinum-based drugs by activating DNA repair pathways^26^; However, it can enhance sensitivity to antimetabolites such as 5-fluorouracil by interfering with nucleotide metabolism^30^. Third, regulation by the tumor microenvironment: Tumor-associated macrophages and other cells can indirectly modulate SPHK1’s regulation of drug sensitivity through the secretion of S1P or cytokines^23^. These findings underscore the complexity of biology and future research will need to employ genetically edited cell models multi-omics analyses, genetically edited cell models, and clinical cohort validations to clarify the specific causal mechanisms and molecular underpinnings of SPHK1’s regulation of drug responses.

The experimental validation showed that our RT-qPCR and IHC results in HNSC, STAD, and LIHC consistently confirmed the coordinated upregulation of SPHK1 at both transcriptional and translational levels. This supports the notion that its overexpression is a stable event in tumors. A positive correlation between mRNA and protein expression exists across different cancer types. This suggests that expression is probably regulated at the transcriptional or pre-transcriptional level. Furthermore, survival analysis also hints at its association with poor prognosis. However, the sample size of 20 pairs per cancer type limits the generalizability of the conclusions; therefore, these results should be regarded as important conceptual validations. Future research requires rigorous multivariate analyses with expanded sample sizes and detailed treatment histories. It also involves designing functional experiments, such as apoptosis assays, migration and invasion assays, analysis of key signaling pathways, and in vivo tumorigenicity experiments. Rescue experiments should be conducted to clarify the specificity of the phenotypes, thereby confirming that SPHK1 is a “driver” rather than a mere “bystander” in tumor progression.

This study has several limitations. First, Retrospective analyses based on public transcriptomic databases are prone to potential biases, therefore, there is a need to integrate multi-omics data and strengthen experimental validation in the future. Secondly, because observational analyses struggle to establish causal relationships, the conclusions require further validation in prospective, large-sample, multicenter cohorts. Lastly, the specific molecular pathways by which SPHK1 functions as a “metabolic immune checkpoint” to regulate tumor immunity remain to be elucidated by further mechanistic studies.

## Conclusions

In summary, this pan-cancer study has elevated SPHK1’s role from a conventional oncogene and prognostic biomarker to a key “metabolic immune checkpoint.” This redefinition provides a fresh perspective on how tumors exploit metabolic reprogramming to orchestrate immune evasion. It also lays a solid theoretical and evidentiary foundation for developing next-generation precision strategies that combine metabolic intervention with immunotherapy.

## Supplementary Information

Below is the link to the electronic supplementary material.

Supplementary Material 1

Supplementary Material 2

Supplementary Material 3

## Data Availability

The data used for bioinformatic analyses during the current study are original from public databases, including The Cancer Genome Atlas (TCGA) (available at https://portal.gdc.cancer.gov/), the Human Protein Atlas (HPA) (accessible at https://www.proteinatlas.org/), The cBioPortal platform (available at https://www.cbioportal.org/), The UALCAN database (http://ualcan.path.uab.edu/), BioGRID (https://thebiogrid.org/), STRING(https://cn.string-db.org/), GDSC (https://www.cancerrxgene.org/), CTRP (https://portals.broadinstitute.org/ctrp/) and CellMiner (https://discover.nci.nih.gov/cellminer/home.do).The data used for the validation experiment are available from the corresponding author upon reasonable request. All scripts and processed data matrices for this study were uploaded to a public GitHub repository accessible via the following link: https://github.com/xhbuestc/SPHK1-pan-cancer-analysis/tree/main/Code; https://github.com/xhbuestc/SPHK1-pan-cancer-analysis/tree/main/Processed%20matrics.

## Abbreviations

ACC: Adrenocortical carcinoma
BLCA: Bladder urothelial carcinoma
BRCA: Breast invasive carcinoma
CESC: Cervical squamous cell carcinoma and endocervical adenocarcinoma
CHOL: Cholangiocarcinoma
COAD: Colon adenocarcinoma
DLBC: Lymphoid neoplasm diffuse large B-cell lymphoma
ESCA: Esophageal carcinoma
GBM: Glioblastoma multiforme
HNSC: Head and neck squamous cell carcinoma
KICH: Kidney chromophobe
KIRC: Kidney renal clear cell carcinoma
KIRP: Kidney renal papillary cell carcinoma
LAML: Acute myeloid leukemia
LGG: Brain lower grade glioma
LIHC: Liver hepatocellular carcinoma
LUAD: Lung adenocarcinoma
LUSC: Lung squamous cell carcinoma
MESO: Mesothelioma
OV: Ovarian serous cystadenocarcinoma
PAAD: Pancreatic adenocarcinoma
PCPG: Pheochromocytoma and paraganglioma
PRAD: Prostate adenocarcinoma
READ: Rectum adenocarcinoma
SARC: Sarcoma
SKCM: Skin cutaneous melanoma
STAD: Stomach adenocarcinoma
TGCT: Testicular germ cell tumors
THCA: Thyroid carcinoma
THYM: Thymoma
UCEC: Uterine corpus endometrial carcinoma
UCS: Uterine carcinosarcoma
UVM: Uveal melanoma
OS: Overall survival
DSS: Disease specific survival
PFI: Progress free interval
GO: Gene ontology
KEGG: Kyoto encyclopedia of genes and genomes
IHC: Immunohistochemistry
ROC: Receiver operating characteristic
GDSC: Genomics of drug sensitivity in cancer
CTRP: The cancer therapeutics response portal
Cor: Correlation coefficient
TCGA: The cancer Genome atlas
