# Supplementary material for: The emerging role of SPHK1 at the immune-metabolic interface: a pan-cancer integrative analysis
Source: Sci Rep. 2026 Jan 17;16:5528. doi: 10.1038/s41598-026-35350-7 (PMC12886843; doi:10.1038/s41598-026-35350-7)
Supplement: Supplementary file 1 — Supplementary Material 1 [file 41598_2026_35350_MOESM1_ESM.docx]

Supplementary Table S1 Univariate and multivariate analysis for overall survival (OS) of ACC patients.

| Characteristics | Total(N) | Univariate analysis | |  | Multivariate analysis | |
| --- | --- | --- | --- | --- | --- | --- |
|  |  | Hazard ratio (95% CI) | P value |  | Hazard ratio (95% CI) | P value |
| Pathologic T stage | 77 |  |  |  |  |  |
| T1&T2 | 51 | Reference |  |  | Reference |  |
| T3&T4 | 26 | 10.286 (3.976 - 26.608) | **< 0.001** |  | 8.973 (1.120 - 71.868) | **0.039** |
| Pathologic N stage | 77 |  |  |  |  |  |
| N0 | 68 | Reference |  |  |  |  |
| N1 | 9 | 2.038 (0.769 - 5.400) | 0.152 |  |  |  |
| SPHK1 | 79 |  |  |  |  |  |
| Low | 39 | Reference |  |  | Reference |  |
| High | 40 | 2.879 (1.299 - 6.380) | **0.009** |  | 2.604 (1.115 - 6.080) | **0.027** |
| Pathologic stage | 77 |  |  |  |  |  |
| Stage I&Stage II | 46 | Reference |  |  | Reference |  |
| Stage III&Stage IV | 31 | 6.476 (2.706 - 15.498) | **< 0.001** |  | 0.782 (0.094 - 6.532) | 0.821 |
| Clinical M stage | 77 |  |  |  |  |  |
| M0 | 62 | Reference |  |  | Reference |  |
| M1 | 15 | 6.150 (2.710 - 13.959) | **< 0.001** |  | 1.650 (0.634 - 4.296) | 0.305 |
| Gender | 79 |  |  |  |  |  |
| Female | 48 | Reference |  |  |  |  |
| Male | 31 | 1.001 (0.469 - 2.137) | 0.999 |  |  |  |
| Age | 79 |  |  |  |  |  |
| <= 50 | 41 | Reference |  |  |  |  |
| > 50 | 38 | 1.799 (0.846 - 3.824) | 0.127 |  |  |  |

Supplementary Table S2 Univariate and multivariate analysis for overall survival (OS) of KIRC patients

| Characteristics | Total(N) | Univariate analysis | |  | Multivariate analysis | |
| --- | --- | --- | --- | --- | --- | --- |
|  |  | Hazard ratio (95% CI) | P value |  | Hazard ratio (95% CI) | P value |
| Pathologic T stage | 541 |  |  |  |  |  |
| T1&T2 | 350 | Reference |  |  | Reference |  |
| T3&T4 | 191 | 3.210 (2.373 - 4.342) | **< 0.001** |  | 1.387 (0.608 - 3.165) | 0.436 |
| Pathologic N stage | 258 |  |  |  |  |  |
| N0 | 242 | Reference |  |  | Reference |  |
| N1 | 16 | 3.422 (1.817 - 6.446) | **< 0.001** |  | 1.549 (0.771 - 3.112) | 0.219 |
| SPHK1 | 541 |  |  |  |  |  |
| Low | 270 | Reference |  |  | Reference |  |
| High | 271 | 1.894 (1.396 - 2.568) | **< 0.001** |  | 1.697 (1.076 - 2.677) | **0.023** |
| Pathologic M stage | 508 |  |  |  |  |  |
| M0 | 429 | Reference |  |  | Reference |  |
| M1 | 79 | 4.401 (3.226 - 6.002) | **< 0.001** |  | 2.502 (1.477 - 4.239) | **< 0.001** |
| Pathologic stage | 538 |  |  |  |  |  |
| Stage I&Stage II | 332 | Reference |  |  | Reference |  |
| Stage III&Stage IV | 206 | 3.910 (2.852 - 5.360) | **< 0.001** |  | 1.354 (0.536 - 3.422) | 0.521 |
| Histologic grade | 533 |  |  |  |  |  |
| G1&G2 | 250 | Reference |  |  | Reference |  |
| G3&G4 | 283 | 2.665 (1.898 - 3.743) | **< 0.001** |  | 1.594 (0.971 - 2.614) | 0.065 |
| Gender | 541 |  |  |  |  |  |
| Female | 187 | Reference |  |  |  |  |
| Male | 354 | 0.924 (0.679 - 1.257) | 0.613 |  |  |  |
| Age | 541 |  |  |  |  |  |
| <= 60 | 269 | Reference |  |  | Reference |  |
| > 60 | 272 | 1.791 (1.319 - 2.432) | **< 0.001** |  | 1.700 (1.110 - 2.602) | **0.015** |

Supplementary Table S3 Univariate and multivariate analysis for overall survival (OS) of LIHC patients.

| Characteristics | Total(N) | Univariate analysis | |  | Multivariate analysis | |
| --- | --- | --- | --- | --- | --- | --- |
|  |  | Hazard ratio (95% CI) | P value |  | Hazard ratio (95% CI) | P value |
| Pathologic T stage | 370 |  |  |  |  |  |
| T1&T2 | 277 | Reference |  |  | Reference |  |
| T3&T4 | 93 | 2.598 (1.826 - 3.697) | **< 0.001** |  | 1.897 (0.257 - 14.003) | 0.530 |
| Pathologic N stage | 258 |  |  |  |  |  |
| N0 | 254 | Reference |  |  |  |  |
| N1 | 4 | 2.029 (0.497 - 8.281) | 0.324 |  |  |  |
| SPHK1 | 373 |  |  |  |  |  |
| Low | 187 | Reference |  |  | Reference |  |
| High | 186 | 1.451 (1.026 - 2.052) | **0.035** |  | 1.564 (1.013 - 2.415) | **0.043** |
| Pathologic M stage | 272 |  |  |  |  |  |
| M0 | 268 | Reference |  |  | Reference |  |
| M1 | 4 | 4.077 (1.281 - 12.973) | **0.017** |  | 1.931 (0.591 - 6.303) | 0.276 |
| Pathologic stage | 349 |  |  |  |  |  |
| Stage I&Stage II | 259 | Reference |  |  | Reference |  |
| Stage III&Stage IV | 90 | 2.504 (1.727 - 3.631) | **< 0.001** |  | 1.464 (0.199 - 10.756) | 0.708 |
| Gender | 373 |  |  |  |  |  |
| Female | 121 | Reference |  |  |  |  |
| Male | 252 | 0.793 (0.557 - 1.130) | 0.200 |  |  |  |
| Age | 373 |  |  |  |  |  |
| <= 60 | 177 | Reference |  |  |  |  |
| > 60 | 196 | 1.205 (0.850 - 1.708) | 0.295 |  |  |  |

Supplementary Table S4 Univariate and multivariate analysis for overall survival (OS) of MESO patients.

| Characteristics | Total(N) | Univariate analysis | |  | Multivariate analysis | |
| --- | --- | --- | --- | --- | --- | --- |
|  |  | Hazard ratio (95% CI) | P value |  | Hazard ratio (95% CI) | P value |
| Pathologic T stage | 84 |  |  |  |  |  |
| T1&T2 | 39 | Reference |  |  |  |  |
| T3&T4 | 45 | 0.978 (0.606 - 1.578) | 0.927 |  |  |  |
| Pathologic N stage | 82 |  |  |  |  |  |
| N0&N1 | 54 | Reference |  |  |  |  |
| N2&N3 | 28 | 0.884 (0.530 - 1.474) | 0.637 |  |  |  |
| SPHK1 | 86 |  |  |  |  |  |
| Low | 43 | Reference |  |  | Reference |  |
| High | 43 | 2.414 (1.492 - 3.904) | **< 0.001** |  | 2.414 (1.492 - 3.904) | **< 0.001** |
| Pathologic M stage | 60 |  |  |  |  |  |
| M0 | 57 | Reference |  |  |  |  |
| M1 | 3 | 1.856 (0.441 - 7.817) | 0.399 |  |  |  |
| Pathologic stage | 86 |  |  |  |  |  |
| Stage I&Stage II | 26 | Reference |  |  |  |  |
| Stage III&Stage IV | 60 | 0.993 (0.588 - 1.675) | 0.978 |  |  |  |
| Gender | 86 |  |  |  |  |  |
| Female | 16 | Reference |  |  |  |  |
| Male | 70 | 0.888 (0.494 - 1.595) | 0.691 |  |  |  |
| Age | 86 |  |  |  |  |  |
| <= 65 | 46 | Reference |  |  |  |  |
| > 65 | 40 | 1.325 (0.826 - 2.125) | 0.243 |  |  |  |

Supplementary Table S5 Univariate and multivariate analysis for overall survival (OS) of UVM patients.

| Characteristics | Total(N) | Univariate analysis | |  | Multivariate analysis | |
| --- | --- | --- | --- | --- | --- | --- |
|  |  | Hazard ratio (95% CI) | P value |  | Hazard ratio (95% CI) | P value |
| Pathologic M stage | 78 |  |  |  |  |  |
| M0 | 51 | Reference |  |  |  |  |
| M1&MX | 27 | 0.924 (0.373 - 2.287) | 0.865 |  |  |  |
| Pathologic stage | 79 |  |  |  |  |  |
| Stage II | 39 | Reference |  |  |  |  |
| Stage III&Stage IV | 40 | 1.502 (0.629 - 3.585) | 0.360 |  |  |  |
| SPHK1 | 80 |  |  |  |  |  |
| Low | 40 | Reference |  |  | Reference |  |
| High | 40 | 4.144 (1.522 - 11.283) | **0.005** |  | 4.011 (1.467 - 10.966) | **0.007** |
| Clinical T stage | 78 |  |  |  |  |  |
| T2 | 4 | Reference |  |  |  |  |
| T3&T4 | 74 | 75584651.8840 (0.000 - Inf) | 0.998 |  |  |  |
| Clinical N stage | 80 |  |  |  |  |  |
| N0 | 76 | Reference |  |  | Reference |  |
| NX | 4 | 6.177 (1.302 - 29.304) | **0.022** |  | 3.866 (0.480 - 31.118) | 0.204 |
| Clinical M stage | 80 |  |  |  |  |  |
| M0 | 73 | Reference |  |  | Reference |  |
| M1&MX | 7 | 4.004 (1.321 - 12.140) | **0.014** |  | 3.122 (0.630 - 15.466) | 0.163 |
| Clinical stage | 80 |  |  |  |  |  |
| Stage II | 36 | Reference |  |  |  |  |
| Stage III&Stage IV | 44 | 1.718 (0.704 - 4.193) | 0.234 |  |  |  |
| Gender | 80 |  |  |  |  |  |
| Female | 35 | Reference |  |  |  |  |
| Male | 45 | 1.542 (0.651 - 3.652) | 0.325 |  |  |  |
| Age | 80 |  |  |  |  |  |
| <= 60 | 40 | Reference |  |  | Reference |  |
| > 60 | 40 | 2.123 (0.914 - 4.933) | 0.080 |  | 3.228 (1.229 - 8.478) | **0.017** |

Supplementary Table S6 Univariate and multivariate analysis for disease specific survival (DSS) of ACC patients.

| Characteristics | Total(N) | Univariate analysis | |  | Multivariate analysis | |
| --- | --- | --- | --- | --- | --- | --- |
|  |  | Hazard ratio (95% CI) | P value |  | Hazard ratio (95% CI) | P value |
| Pathologic T stage | 76 |  |  |  |  |  |
| T1&T2 | 51 | Reference |  |  | Reference |  |
| T3&T4 | 25 | 9.927 (3.812 - 25.853) | **< 0.001** |  | 8.767 (1.091 - 70.430) | **0.041** |
| Pathologic N stage | 76 |  |  |  |  |  |
| N0 | 67 | Reference |  |  |  |  |
| N1 | 9 | 2.115 (0.795 - 5.630) | 0.134 |  |  |  |
| SPHK1 | 77 |  |  |  |  |  |
| Low | 38 | Reference |  |  | Reference |  |
| High | 39 | 2.979 (1.293 - 6.867) | **0.010** |  | 2.549 (1.087 - 5.980) | **0.031** |
| Pathologic stage | 76 |  |  |  |  |  |
| Stage I&Stage II | 46 | Reference |  |  | Reference |  |
| Stage III&Stage IV | 30 | 6.256 (2.596 - 15.079) | **< 0.001** |  | 0.795 (0.095 - 6.636) | 0.832 |
| Clinical M stage | 76 |  |  |  |  |  |
| M0 | 62 | Reference |  |  | Reference |  |
| M1 | 14 | 5.880 (2.549 - 13.568) | **< 0.001** |  | 1.614 (0.610 - 4.269) | 0.335 |
| Gender | 77 |  |  |  |  |  |
| Female | 48 | Reference |  |  |  |  |
| Male | 29 | 0.869 (0.387 - 1.950) | 0.733 |  |  |  |
| Age | 77 |  |  |  |  |  |
| <= 50 | 40 | Reference |  |  |  |  |
| > 50 | 37 | 1.785 (0.816 - 3.906) | 0.147 |  |  |  |

Supplementary Table S7 Univariate and multivariate analysis for disease specific survival (DSS) of KIRC patients.

| Characteristics | Total(N) | Univariate analysis | |  | Multivariate analysis | |
| --- | --- | --- | --- | --- | --- | --- |
|  |  | Hazard ratio (95% CI) | P value |  | Hazard ratio (95% CI) | P value |
| Pathologic T stage | 530 |  |  |  |  |  |
| T1&T2 | 347 | Reference |  |  | Reference |  |
| T3&T4 | 183 | 5.606 (3.697 - 8.502) | **< 0.001** |  | 1.025 (0.440 - 2.389) | 0.954 |
| Pathologic N stage | 256 |  |  |  |  |  |
| N0 | 241 | Reference |  |  | Reference |  |
| N1 | 15 | 3.864 (1.831 - 8.157) | **< 0.001** |  | 1.285 (0.593 - 2.784) | 0.526 |
| SPHK1 | 530 |  |  |  |  |  |
| Low | 261 | Reference |  |  | Reference |  |
| High | 269 | 2.775 (1.836 - 4.193) | **< 0.001** |  | 2.225 (1.205 - 4.107) | **0.011** |
| Pathologic M stage | 497 |  |  |  |  |  |
| M0 | 422 | Reference |  |  | Reference |  |
| M1 | 75 | 9.219 (6.294 - 13.504) | **< 0.001** |  | 3.141 (1.746 - 5.650) | **< 0.001** |
| Pathologic stage | 527 |  |  |  |  |  |
| Stage I&Stage II | 329 | Reference |  |  | Reference |  |
| Stage III&Stage IV | 198 | 9.937 (5.989 - 16.486) | **< 0.001** |  | 3.629 (1.216 - 10.831) | **0.021** |
| Histologic grade | 522 |  |  |  |  |  |
| G1&G2 | 249 | Reference |  |  | Reference |  |
| G3&G4 | 273 | 4.850 (2.925 - 8.043) | **< 0.001** |  | 1.922 (0.979 - 3.775) | 0.058 |
| Gender | 530 |  |  |  |  |  |
| Female | 181 | Reference |  |  |  |  |
| Male | 349 | 1.183 (0.786 - 1.781) | 0.420 |  |  |  |
| Age | 530 |  |  |  |  |  |
| <= 60 | 265 | Reference |  |  |  |  |
| > 60 | 265 | 1.351 (0.926 - 1.971) | 0.118 |  |  |  |

Supplementary Table S8 Univariate and multivariate analysis for disease specific survival (DSS) of MESO patients

| Characteristics | Total(N) | Univariate analysis | |  | Multivariate analysis | |
| --- | --- | --- | --- | --- | --- | --- |
|  |  | Hazard ratio (95% CI) | P value |  | Hazard ratio (95% CI) | P value |
| Pathologic T stage | 65 |  |  |  |  |  |
| T1&T2 | 32 | Reference |  |  |  |  |
| T3&T4 | 33 | 1.190 (0.643 - 2.205) | 0.580 |  |  |  |
| Pathologic N stage | 62 |  |  |  |  |  |
| N0&N1 | 41 | Reference |  |  |  |  |
| N2&N3 | 21 | 0.773 (0.397 - 1.504) | 0.448 |  |  |  |
| SPHK1 | 66 |  |  |  |  |  |
| Low | 36 | Reference |  |  | Reference |  |
| High | 30 | 2.115 (1.156 - 3.872) | **0.015** |  | 2.115 (1.156 - 3.872) | **0.015** |
| Pathologic M stage | 54 |  |  |  |  |  |
| M0 | 51 | Reference |  |  |  |  |
| M1 | 3 | 2.327 (0.541 - 10.007) | 0.257 |  |  |  |
| Pathologic stage | 66 |  |  |  |  |  |
| Stage I&Stage II | 22 | Reference |  |  |  |  |
| Stage III&Stage IV | 44 | 0.832 (0.436 - 1.587) | 0.576 |  |  |  |
| Gender | 66 |  |  |  |  |  |
| Female | 15 | Reference |  |  |  |  |
| Male | 51 | 0.843 (0.424 - 1.676) | 0.626 |  |  |  |
| Age | 66 |  |  |  |  |  |
| <= 65 | 34 | Reference |  |  |  |  |
| > 65 | 32 | 1.031 (0.563 - 1.887) | 0.922 |  |  |  |

Supplementary Table S9 Univariate and multivariate analysis for disease specific survival (DSS) of UVM patients

| Characteristics | Total(N) | Univariate analysis | |  | Multivariate analysis | |
| --- | --- | --- | --- | --- | --- | --- |
|  |  | Hazard ratio (95% CI) | P value |  | Hazard ratio (95% CI) | P value |
| Pathologic M stage | 78 |  |  |  |  |  |
| M0 | 51 | Reference |  |  |  |  |
| M1&MX | 27 | 1.024 (0.405 - 2.585) | 0.961 |  |  |  |
| Pathologic stage | 79 |  |  |  |  |  |
| Stage II | 39 | Reference |  |  |  |  |
| Stage III&Stage IV | 40 | 1.607 (0.640 - 4.033) | 0.312 |  |  |  |
| SPHK1 | 80 |  |  |  |  |  |
| Low | 40 | Reference |  |  | Reference |  |
| High | 40 | 4.547 (1.529 - 13.525) | **0.006** |  | 3.722 (1.217 - 11.386) | **0.021** |
| Clinical T stage | 78 |  |  |  |  |  |
| T2 | 4 | Reference |  |  |  |  |
| T3&T4 | 74 | 72582838.3852 (0.000 - Inf) | 0.998 |  |  |  |
| Clinical N stage | 80 |  |  |  |  |  |
| N0 | 76 | Reference |  |  | Reference |  |
| NX | 4 | 6.177 (1.302 - 29.304) | **0.022** |  | 5.254 (0.572 - 48.235) | 0.142 |
| Clinical M stage | 80 |  |  |  |  |  |
| M0 | 73 | Reference |  |  | Reference |  |
| M1&MX | 7 | 4.095 (1.345 - 12.471) | **0.013** |  | 1.541 (0.331 - 7.175) | 0.582 |
| Clinical stage | 80 |  |  |  |  |  |
| Stage II | 36 | Reference |  |  | Reference |  |
| Stage III&Stage IV | 44 | 2.318 (0.848 - 6.337) | 0.101 |  | 2.018 (0.678 - 6.005) | 0.207 |
| Gender | 80 |  |  |  |  |  |
| Female | 35 | Reference |  |  |  |  |
| Male | 45 | 1.351 (0.558 - 3.275) | 0.505 |  |  |  |
| Age | 80 |  |  |  |  |  |
| <= 60 | 40 | Reference |  |  |  |  |
| > 60 | 40 | 1.872 (0.785 - 4.461) | 0.157 |  |  |  |

Supplementary Table S10 Univariate and multivariate analysis for progress free interval (PFI) of DLBC patients

| Characteristics | Total(N) | Univariate analysis | |  | Multivariate analysis | |
| --- | --- | --- | --- | --- | --- | --- |
|  |  | Hazard ratio (95% CI) | P value |  | Hazard ratio (95% CI) | P value |
| Clinical stage | 42 |  |  |  |  |  |
| Stage I&Stage II | 25 | Reference |  |  | Reference |  |
| Stage III&Stage IV | 17 | 3.486 (0.894 - 13.589) | 0.072 |  | 1.812 (0.316 - 10.384) | 0.505 |
| SPHK1 | 48 |  |  |  |  |  |
| Low | 24 | Reference |  |  | Reference |  |
| High | 24 | 0.180 (0.038 - 0.845) | **0.030** |  | 0.150 (0.027 - 0.822) | **0.029** |
| Gender | 48 |  |  |  |  |  |
| Female | 26 | Reference |  |  |  |  |
| Male | 22 | 0.354 (0.092 - 1.366) | 0.132 |  |  |  |
| Age | 48 |  |  |  |  |  |
| <= 60 | 27 | Reference |  |  |  |  |
| > 60 | 21 | 1.906 (0.574 - 6.334) | 0.292 |  |  |  |
| BMI | 48 |  |  |  |  |  |
| <= 25 | 25 | Reference |  |  |  |  |
| > 25 | 23 | 0.803 (0.241 - 2.678) | 0.721 |  |  |  |
| Primary therapy outcome | 46 |  |  |  |  |  |
| PD&SD | 8 | Reference |  |  | Reference |  |
| PR&CR | 38 | 0.096 (0.025 - 0.367) | **< 0.001** |  | 0.078 (0.013 - 0.473) | **0.006** |
| Extranodal involvement | 46 |  |  |  |  |  |
| No | 25 | Reference |  |  |  |  |
| Yes | 21 | 0.766 (0.223 - 2.635) | 0.673 |  |  |  |

Supplementary Table S11 Univariate and multivariate analysis for progress free interval (PFI) of UVM patients

| Characteristics | Total(N) | Univariate analysis | |  | Multivariate analysis | |
| --- | --- | --- | --- | --- | --- | --- |
|  |  | Hazard ratio (95% CI) | P value |  | Hazard ratio (95% CI) | P value |
| Pathologic M stage | 77 |  |  |  |  |  |
| M0 | 51 | Reference |  |  |  |  |
| M1&MX | 26 | 1.298 (0.585 - 2.878) | 0.521 |  |  |  |
| Pathologic stage | 78 |  |  |  |  |  |
| Stage II | 39 | Reference |  |  |  |  |
| Stage III&Stage IV | 39 | 1.398 (0.654 - 2.987) | 0.387 |  |  |  |
| SPHK1 | 79 |  |  |  |  |  |
| Low | 40 | Reference |  |  | Reference |  |
| High | 39 | 2.856 (1.245 - 6.554) | **0.013** |  | 2.936 (1.276 - 6.759) | **0.011** |
| Clinical T stage | 77 |  |  |  |  |  |
| T2 | 4 | Reference |  |  |  |  |
| T3&T4 | 73 | 0.381 (0.088 - 1.652) | 0.197 |  |  |  |
| Clinical N stage | 79 |  |  |  |  |  |
| N0 | 75 | Reference |  |  |  |  |
| NX | 4 | 4.133 (0.923 - 18.516) | 0.064 |  |  |  |
| Clinical M stage | 79 |  |  |  |  |  |
| M0 | 73 | Reference |  |  | Reference |  |
| M1&MX | 6 | 8.254 (2.548 - 26.736) | **< 0.001** |  | 8.936 (2.659 - 30.032) | **< 0.001** |
| Clinical stage | 79 |  |  |  |  |  |
| Stage II | 36 | Reference |  |  |  |  |
| Stage III&Stage IV | 43 | 1.619 (0.748 - 3.507) | 0.222 |  |  |  |
| Gender | 79 |  |  |  |  |  |
| Female | 35 | Reference |  |  |  |  |
| Male | 44 | 0.820 (0.393 - 1.710) | 0.597 |  |  |  |
| Age | 79 |  |  |  |  |  |
| <= 60 | 40 | Reference |  |  |  |  |
| > 60 | 39 | 1.387 (0.663 - 2.902) | 0.386 |  |  |  |
