# Supplementary material for: The emerging role of SPHK1 at the immune-metabolic interface: a pan-cancer integrative analysis
Source: Sci Rep. 2026 Jan 17;16:5528. doi: 10.1038/s41598-026-35350-7 (PMC12886843; doi:10.1038/s41598-026-35350-7)
Supplement: Supplementary file 2 — Supplementary Material 2 [file 41598_2026_35350_MOESM2_ESM.docx]

Supplementary Table S12 Baseline charateristics of patients with HNSC, LIHC and STAD.

| Cancer type | Characteristics | Low expression of SPHK1 | High expression of SPHK1 |
| --- | --- | --- | --- |
| HNSC | n | 10 | 10 |
|  | Pathologic T stage, n (%) |  |  |
|  | T1 | 2 (10%) | 3 (15%) |
|  | T_2-4_ | 8 (40%) | 7 (35%) |
|  | Pathologic N stage, n (%) |  |  |
|  | N0 | 4 (20%) | 5 (25%) |
|  | N_1-3_ | 6 (30%) | 5 (25%) |
|  | Gender, n (%) |  |  |
|  | Female | 3 (15%) | 3 (15%) |
|  | Male | 7 (35%) | 7 (35%) |
|  | Age, n (%) |  |  |
|  | <= 60 | 4 (20%) | 5 (25%) |
|  | > 60 | 6 (30%) | 5 (25%) |
|  | **Histologic grade, n (%)** |  |  |
|  | G_1_ | 4 (20%) | 2 (10%) |
|  | G_2-4_ | 6 (30%) | 8 (40%) |
|  | Alcohol history, n (%) |  |  |
|  | No | 4 (20%) | 3 (15%) |
|  | Yes | 6 (30%) | 7 (35%) |
|  | Smoker, n (%) |  |  |
|  | No | 2 (10%) | 3 (15%) |
|  | Yes | 8 (40%) | 7 (35%) |
|  | Radiation therapy, n (%) |  |  |
|  | No | 3 (15%) | 2 (10%) |
|  | Yes | 7 (35%) | 8 (40%) |
|  | Lymphnode neck dissection, n (%) |  |  |
|  | No | 3 (15%) | 2 (10%) |
|  | Yes | 7 (35%) | 8 (40%) |

| LIHC | n | 10 | 10 |  |
| --- | --- | --- | --- | --- |
|  | Pathologic T stage, n (%) |  |  |  |
|  | T_1_ | 6 (30 %) | 4 (20%) |  |
|  | T_2-4_ | 4 (20 %) | 6 (30%) |  |
|  | Pathologic N stage, n (%) |  |  |  |
|  | N_0_ | 9 (45%) | 8 (40%) |  |
|  | N_1_ | 1 (5%) | 2 (10%) |  |
|  | Gender, n (%) |  |  |  |
|  | Female | 4 (20%) | 3 (15%) |  |
|  | Male | 6 (30%) | 7 (35%) |  |
|  | Age, n (%) |  |  |  |
|  | <= 60 | 3 (15%) | 4 (20%) |  |
|  | > 60 | 7 (35%) | 6 (30%) |  |
|  | Histologic grade, n (%) |  |  |  |
|  | G_1_ | 2 (10%) | 1 (5%) |  |
|  | G_2-4_ | 8 (40%) | 9 (45%) |  |
|  | AFP(ng/ml), n (%) |  |  |  |
|  | <= 400 | 8 (40%) | 6 (30%) |  |
|  | > 400 | 2 (10%) | 4 (20%) |  |

| STAD | n | 10 | 10 |  |
| --- | --- | --- | --- | --- |
|  | Pathologic T stage, n (%) |  |  |  |
|  | T_1_ | 1 (5%) | 2 (10%) |  |
|  | T_2-4_ | 9 (45%) | 8 (40%) |  |
|  | Pathologic N stage, n (%) |  |  |  |
|  | N0 | 4 (20%) | 3 (15%) |  |
|  | N_1-3_ | 6 (30%) | 7 (35%) |  |
|  | Gender, n (%) |  |  |  |
|  | Female | 3 (15%) | 4 (20%) |  |
|  | Male | 7 (35%) | 6 (30%) |  |
|  | Age, n (%) |  |  |  |
|  | <= 65 | 3 (15%) | 4 (20%) |  |
|  | > 65 | 7 (35%) | 6 (30%) |  |
|  | Histologic grade, n (%) |  |  |  |
|  | G_1_ | 1 (5%) | 1 (5%) |  |
|  | G_2-3_ | 9 (45%) | 9 (45%) |  |
|  | Reflux history, n (%) |  |  |  |
|  | No | 8 (40%) | 7 (35%) |  |
|  | Yes | 2 (10%) | 3 (15%) |  |
