# Supplementary material for: The emerging role of SPHK1 at the immune-metabolic interface: a pan-cancer integrative analysis
Source: Sci Rep. 2026 Jan 17;16:5528. doi: 10.1038/s41598-026-35350-7 (PMC12886843; doi:10.1038/s41598-026-35350-7)

**Supplementary Fig. S1** SPHK1 mRNA expression was significantly correlated with pathological stage of ACC, BLAC, CESC, KIRC, LUAD, MESO, TGCT, THCA, and UCEC. *P<0.05; **P<0.01; ***P<0.001.


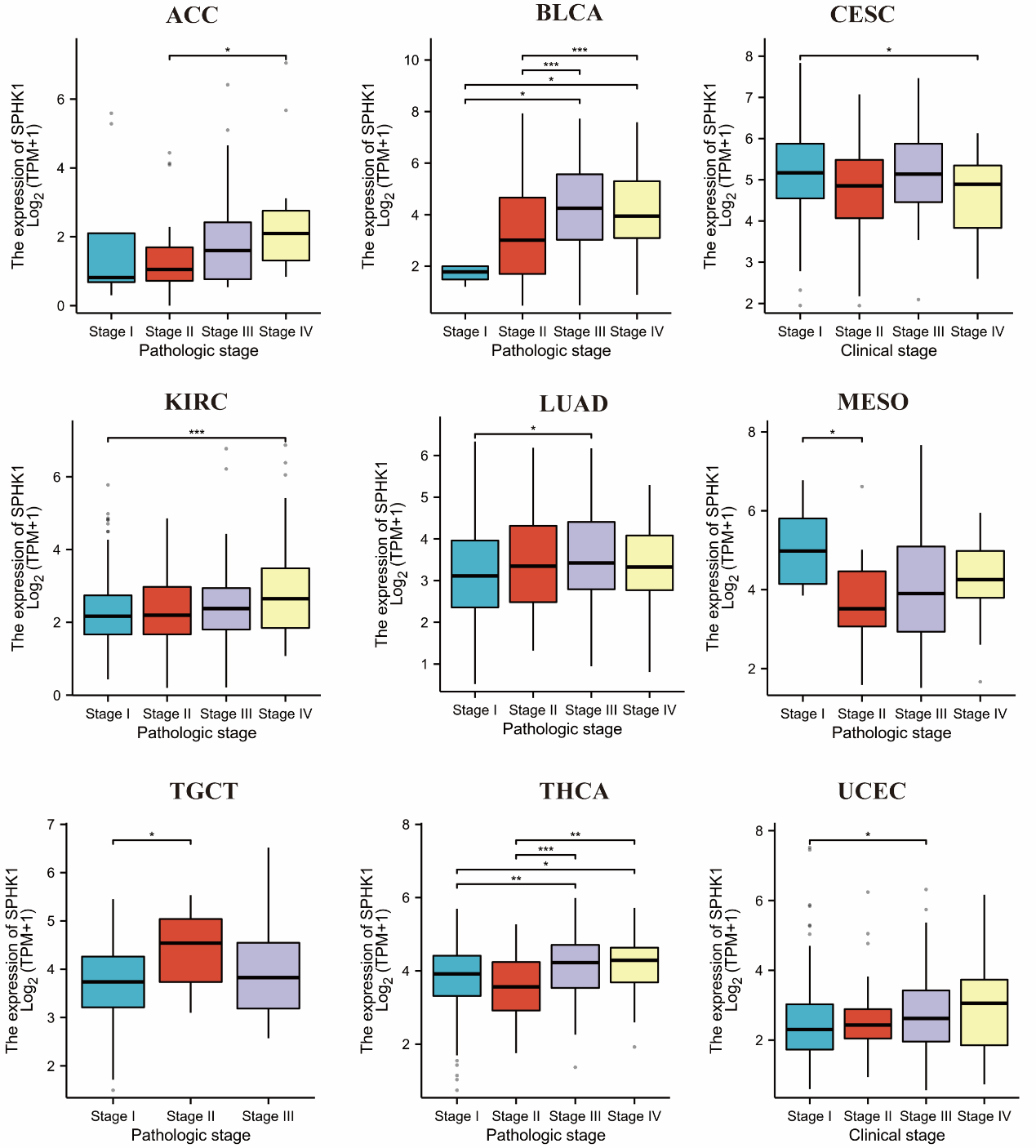


**Supplementary Fig. S2** Mutation features of SPHK1 in various cancers based on TCGA. (A). Alteration frequency of SPHK1 in pan-cancer from cBioPortal website. (B) The 3D structure of mutation site (R191H). (C) Mutation sites of SPHK1 in pan-cancer.


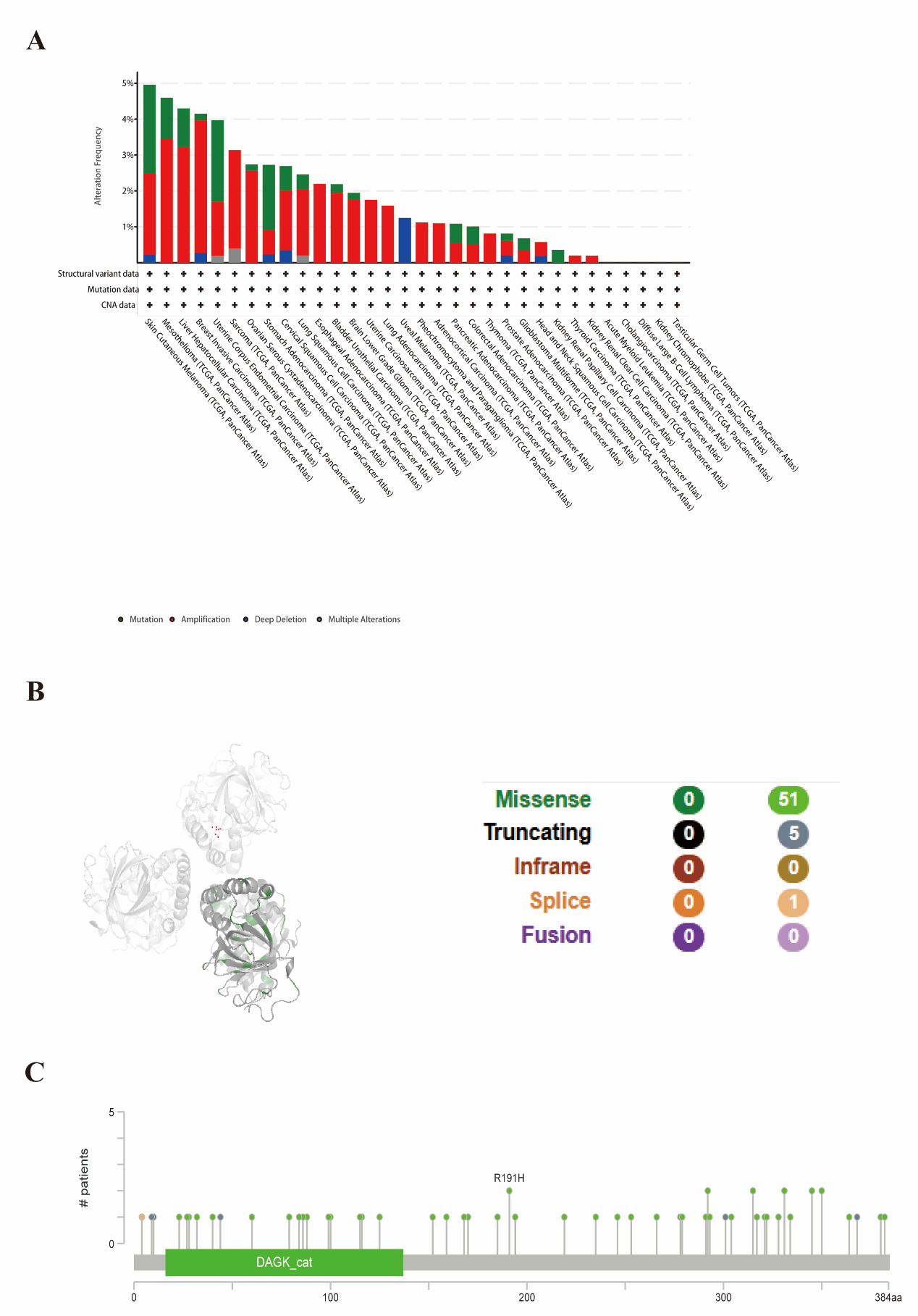


**Supplementary Fig. S3** The enriched signaling pathways of SPHK1 from GSEA in HNSC, STAD and LIHC. (A) The top five enriched up-regulated pathways for HNSC. (B) The top three enriched up-regulated and two down-regulated pathways for STAD. (C) The top five enriched down-regulated pathways for LIHC.


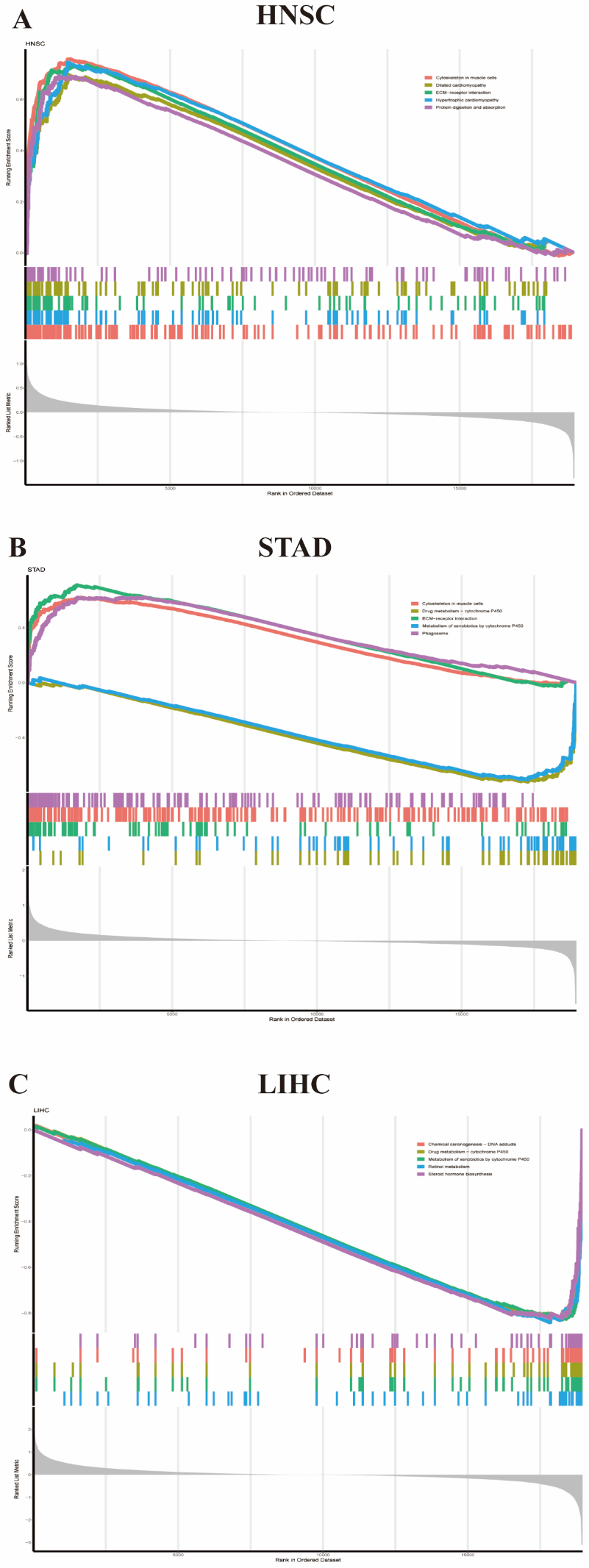


**Supplementary Fig. S4** High SPHK1 protein expression is significantly associated with poorer overall survival in HNSC (A), STAD (B), LIHC (C). SPHK1 mRNA was significantly positive correlated with protein levels in HNSC (D), STAD (E), and LIHC (F).


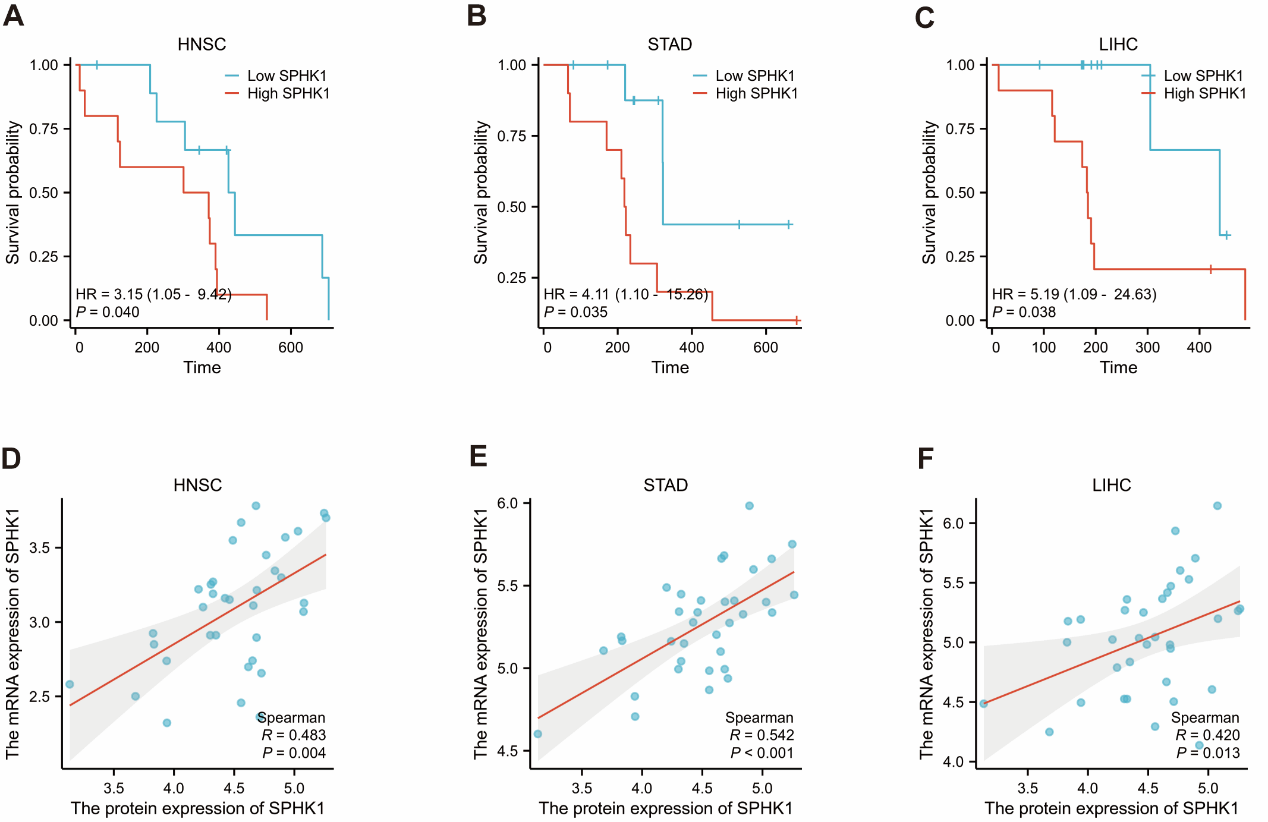


**Supplementary Fig. S5** SPHK1 expression in HNSC (GSE107591), STAD (GSE26942), and LIHC (GSE57957) datasets from the GEO database were higher in tumor tissues than in adjacent or normal tissues.


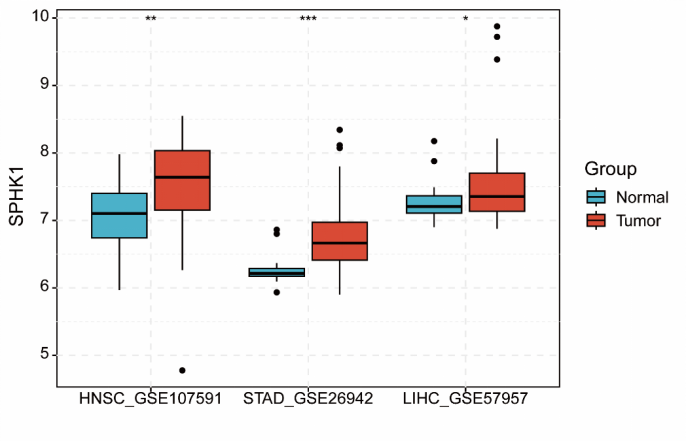

Supplement: Supplementary file 3 — Supplementary Material 3 [file 41598_2026_35350_MOESM3_ESM.docx]
